# A computer model simulating human glucose absorption and metabolism in health and metabolic disease states

**DOI:** 10.12688/f1000research.8299.1

**Published:** 2016-04-12

**Authors:** Richard J. Naftalin

**Affiliations:** 1 Departments of Physiology and Vascular Biology, BHF centre of research excellence, King's College London School of Medicine, London, UK

**Keywords:** Simulation of human intestinal glucose absorption, GLP-1, Insulin, Glucagon, Superior mesenteric arterial blood flow, Non-alcoholic steatohepatitis, Type 2 diabetes, Portosystemic shunting, Hyperinsulinaemia, Hyperglucagonaemia.

## Abstract

A computer model designed to simulate integrated glucose-dependent changes in splanchnic blood flow with small intestinal glucose absorption, hormonal and incretin circulation and hepatic and systemic metabolism in health and metabolic diseases e.g. non-alcoholic fatty liver disease, (NAFLD), non-alcoholic steatohepatitis, (NASH) and type 2 diabetes mellitus, (T2DM) demonstrates how when glucagon-like peptide-1, (GLP-1) is synchronously released into the splanchnic blood during intestinal glucose absorption, it stimulates superior mesenteric arterial (SMA) blood flow and by increasing passive intestinal glucose absorption, harmonizes absorption with its distribution and metabolism. GLP-1 also synergises insulin-dependent net hepatic glucose uptake (NHGU). When GLP-1 secretion is deficient post-prandial SMA blood flow is not increased and as NHGU is also reduced, hyperglycaemia follows. Portal venous glucose concentration is also raised, thereby retarding the passive component of intestinal glucose absorption.

Increased pre-hepatic sinusoidal resistance combined with portal hypertension leading to opening of intrahepatic portosystemic collateral vessels are NASH-related mechanical defects that alter the balance between splanchnic and systemic distributions of glucose, hormones and incretins.The model reveals the latent contribution of portosystemic shunting in development of metabolic disease. This diverts splanchnic blood content away from the hepatic sinuses to the systemic circulation, particularly during the glucose absorptive phase of digestion, resulting in inappropriate increases in insulin-dependent systemic glucose metabolism.  This hastens onset of hypoglycaemia and thence hyperglucagonaemia. The model reveals that low rates of GLP-1 secretion, frequently associated with T2DM and NASH, may be also be caused by splanchnic hypoglycaemia, rather than to intrinsic loss of incretin secretory capacity. These findings may have therapeutic implications on GLP-1 agonist or glucagon antagonist usage.

## Abbreviations

AMP, adenosine monophosphate; AMPK, adenosine monophosphate–activated kinase; Blood vessel resistance, Hg.s ml ^-1^; C, compartmental volume compliance; GLP-1, glucagon-like peptide-1; GLUT2, low affinity passive glucose transporter type 2 expressed in intestine, liver and pancreatic beta cells; HA, hepatic artery; HOMA, homeostasis model assessment; HV, hepatic vein; Intestinal paracellular glucose permeability, P _gl_; half maximal concentration, K _½_; KO, genetically mutated knock out; L-M method, Levenberg-Marquardt of non-linear least square regression; NASH, non-alcoholic steatohepatitis; Net hepatic glucose uptake, NHGU; OGTT, Oral glucose tolerance test; PV, Portal vein; PSS R, portosystemic shunt resistance; ΔP, Pressure gradients, mm Hg; SGLT1, sodium dependent glucose transporter; SMA, superior mesenteric artery; superior mesenteric capillary, SM cap; Type 2 diabetes mellitus, T2DM; V _max_ maximal velocity.

## Introduction

### The roles of apical SGLT1 and GLUT2 intestinal glucose absorption

The sodium dependent glucose transporter SGLT1 is the only active component of intestinal transport sugar absorption. When SGLT1 is deficient, as in glucose-galactose malabsorption syndrome ^1– 3^, or inactivated by specific inhibitors, such as phloridzin, or similarly acting high efficacy inhibitors e.g. GSK1614235 ^4^, small intestinal sugar absorption is blocked and the ingested sugar load is relegated to the large intestine where it becomes subject to fermentation processes.

It has been argued that exposure to high intestinal luminal glucose concentrations ≥ 15mM, or more modest glucose loads, supplemented with artificial sweeteners, induces small intestinal apical membrane passive glucose transport via GLUT2 ^5, 6^. This process is stimulated by enterocyte AMP kinase(AMPK), triggered by opening of Cav 1.3 Ca ^2+^ channels following SGLT1-dependent depolarization of the apical membrane potential ^7^. However, whether apical GLUT2 has any functional role in net glucose absorption has been questioned. No discernible effect on net intestinal glucose absorption *in vivo* is observed in GLUT2 knock out, (KO) mice, ^8^.

Glucose absorption can only be enhanced by apical GLUT2, when the enterocyte and submucosal glucose concentrations are lower than in the intestinal lumen. The time required to reach steady state glucose accumulation within the enterocytes *in vitro* following exposure is ≤ 2 min ^9, 10^ and within 5 to 10 minutes in vascularly perfused frog ^11^. As net glucose transport across the basolateral membranes is entirely due to passive processes, it follows that this can only occur when intracellular glucose exceeds the submucosal concentrations. Estimates of enterocyte glucose concentrations that are lower than that within the submucosa have been reported ^12^, but as glucose accumulation only occurs in a small proportion of the enterocytes within the intestinal villus, are ascribable to overestimates of the compartmental volume into which glucose is actively accumulated; ^13^. When the intestinal luminal glucose is lower than the enterocyte concentration, any apical component for passive glucose absorption, such as GLUT2, will hinder, rather than assist, net absorption ^14^.

The experimental evidence supporting the accelerant role of apical GLUT2 in glucose uptake is based on data obtained with pharmacological concentrations of inhibitors, such as phloretin and cytochalasin B. These agents have multiple inhibitory effects, on glucose, Cl ^-^, urea and water permeability. When phloridzin is already present, additional high phloretin concentrations may further inhibit any residual SGLT1 glucose transport activity ^1^ and also prevent paracellular sugar absorption by blocking solvent drag effects ^14, 15^. Additionally, any of the pro-absorptive roles of apical GLUTs seen with phloridzin present will be artificially enhanced by the depressed cytosolic glucose concentration ^14^.

### Paracellular glucose absorption

When the intestinal luminal glucose concentration is higher than mesenteric capillary glucose concentration transcellular glucose transport may be supplemented, by passive flow via paracellular routes from the intestinal lumen ^16– 19^. With luminal glucose concentration > 15 mM the passive transport mode becomes predominant. A variable paracellular sugar permeability explains the non-saturable nature of intestinal glucose transport over a concentration range from 15mM to 100mM ^20, 21^ and how ingested ligands that are not transported via either SGLT1 or GLUT2, e.g. rhamnose, L-glucose, or mannitol, rapidly appear in human urine. Paracellular shunts also explain why molecules show size selectivity of transepithelial flows, ^22– 24^ and how inflammatory intestinal diseases, known to loosen intercellular junctions ^25, 26^ induce large increases probe entry into both plasma and urine ^27^.

The highest rates of glucose transport obtained in exercising dogs are more than an order of magnitude higher than those obtained *in vitro* ^20, 28^. *In vitro* experimentation on isolated intestine or intestinal tissue or cells, which has become the normal mode of investigation of intestinal absorption, necessarily removes the intestinal capillary network. This capillary plexus provides the essential bridging component between the proximal sugar absorptive process and its distribution to the splanchnic and systemic circulations. So when it is removed, the major part of the sugar absorption control system is destroyed ^20, 29– 31^. It is evident that lack of capillary perfusion of *in vitro* intestine heavily masks optimal absorptive performance ^21, 32^.

## Integration of intestinal glucose absorption with splanchnic circulation

### Superior mesenteric artery and incretins

The discovery that oral glucose generates a more rapid and larger metabolic response to insulin than equivalent amounts of intravenous glucose suggested that substances secreted by the gut wall during glucose absorption augment insulin release from pancreatic islets and its activity on liver and muscle ^33– 36^. It was inferred that a portal venous signal raises hepatic glucose uptake and stimulates hepatic glycogen synthesis, independent of a rise in insulin.

The superior mesenteric artery (SMA) supplies 600–1800 ml min ^-1^ blood to the glucose absorptive portion of the proximal small intestine ( Figure 2A and 2B). When ingested glucose is present in the intestinal lumen, splanchnic capillaries channel the absorbate via the portal vein to the liver. Splanchnic blood has approximately double the concentrations of absorbed materials and also of pancreatic hormones and incretins that are present in the systemic circulation ( 37, 38; Figure 3E–G).

## An integrated model of glucose transport and metabolism

It is evident that the incretin response to luminal, submucosal and splanchnic venular glucose; the pancreatic islet secretory response to systemic glucose; the hepatic response to incretins; the intrahepatic circulatory responses to portal blood pressure and the systemic metabolic responses to systemic blood concentrations of glucose, insulin and incretins are interrelated and interdependent ^39– 41^.

A quantitative model of the integrated response to glucose ingestion is both lacking and needed to assimilate the extent to which the incretin response to intestinal glucose load affects the balance between splanchnic–systemic blood flow and hepatic and peripheral glucose metabolism. Although there are several compartmental models that simulate intestinal glucose absorption and its subsequent metabolism by liver, none take account of the altered splanchnic blood flows that accompany and accommodate glucose absorption. These models assume that the splanchnic blood compartment imposes no impediment to flows into the liver ^42– 44^. As will be seen from the simulations here, the GLP-1 controlled flows of SMA are an important component in glucose absorption.

Other models, based mainly on the work of Cherrington’s and Bergman’s laboratories, ^45, 46^ give predictive indices of glucose metabolism and insulin-sensitivity in humans with normal and diabetic metabolism. The HOMA model of whole body glucose metabolism in relation to insulin secretion ^47, 48^ accounts for the hepatic contribution to homeostatic control of plasma glucose, but lacks an account of the incretin response, or splanchnic flow response to glucose ingestion, or how hepatic steatosis and/or portal-systemic venous shunting affect these responses. These issues are addressed by the current model.

## Methods

**Figure 1.**
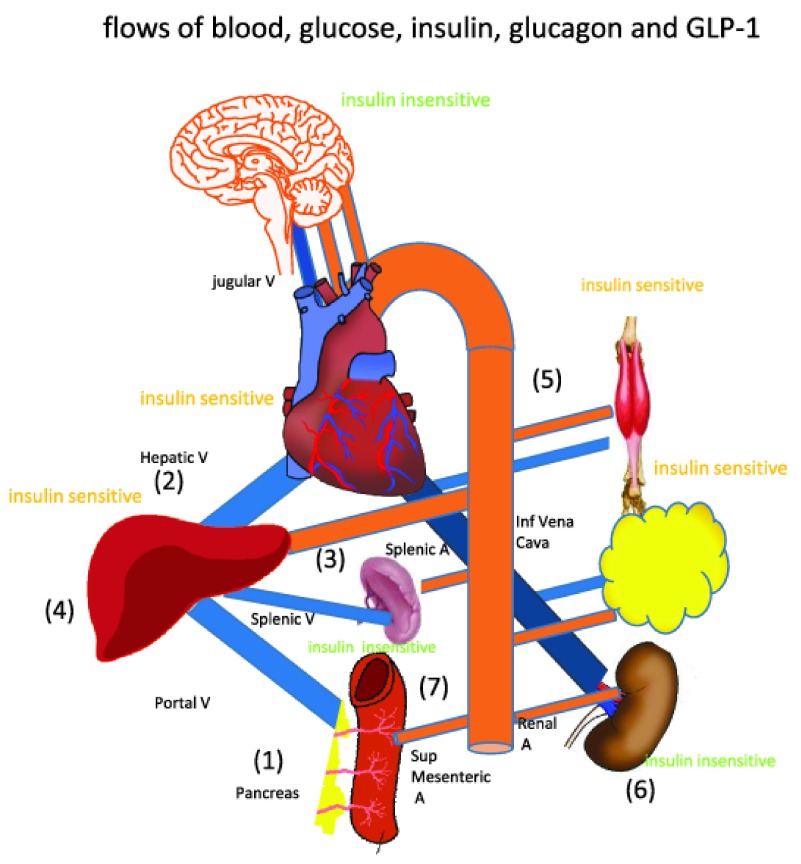
Diagram outlining splanchnic and systemic blood flows.

Replication of the human response to oral glucose ingestion necessitates simulation of the circulatory response to glucose, integrated with hormonal (insulin and glucagon) and incretin (GLP-1) secretion and their effects on the liver and pancreas, also both the peripheral insulin-sensitive (muscle and adipose) tissues and insulin-insensitive (brain, skin and bone) glucose uptakes and metabolism ( Figure 1).

This model of glucose absorption and metabolism was created with several aims. The first was to provide a quantitative simulation of the effects of changes of capillary perfusion rates on intestinal glucose absorption in health and disease. The second was to provide a broader understanding of how incretins affect the whole body response to glucose. The third aim was to demonstrate how metabolic diseases such as NAFLD, NASH and T2DM alter glucose and uptake and metabolism.

The model of whole body glucose absorption builds on those of Granger and Pappenheimer, ^29, 49^. The salient features of the current model are simulation of resting human systemic and splanchnic blood flows and pressures before, during and after glucose absorption. Sets of sub-models simulating the time course of changes in flows and concentrations of glucose, insulin, glucagon and the incretin GLP-1 following intra-duodenal glucose gavage, are embedded within this circulation model ( Figure 1; *for specific details of the model parameters given in parameter Table 2, see also Table 1)*. Intestinal glucose absorption is simulated here following initiation of a standard glucose tolerance test by duodenal gavage. By-passing the stomach avoids the extra complexities resulting from control of gastric emptying rates. Although these factors are important, they are inessential to the intestinal absorptive and subsequent vascular and metabolic processes ^50^.

**Table 1A.** The number prefixes in equations refer to the positions in Figure 1.

*Where R _SMA(0)_ is the SMA blood flow resistance with GLP-1 concentration = 0; R _SMA(t)_ is the SMA resistance at any time t as variable function* *of GLP-1 _SM cap_ concentration and K _m GLP-1_ is the I.C. _50_ of GLP-1 for the GLP-1 receptors in SMA and the rest of the splanchnic circulation.*
**2**A $\int_{\text{t}=0}^{\text{t}=tx}\frac{d}{dt}\;(Sys\;Vein\;vol)\;=\;renalVflow+SomaticVeinflow+Hepshuntflow-Venacavaflow+HVflow$
$SMAflow\text{ = }\frac{aorticB.P–SMcapP}{R\;SMA}\;and\;R_{SMA}=\;\frac{RSMA*KmGLP}{\text{(}KmGLP+GLPSMA\text{)}}\text{.}$
**2**B *Total blood vol = sys art vol* + *SM cap blood vol* + *hep blood vol* + *splanchnic and splenic blood vol* + *Lung blood vol* + *somatic vol* + + *renal blood vol* + *sys vein vol.*
***4*** $\int_{\text{t}=0}^{\text{t}=tx}\frac{d}{dt}\text{(}hepbloodvol\text{)= -}HVflow+HAflow+PVflow+SP\&CeVflows$ ***5*** $\int_{t=0}^{t=tx}\frac{d}{dt}(sysartvolume)=\;aorticflow-SplenicAflow-SMAflow–HAFlow–RenalAflow–(muscleandfatandbrainflows)$ ***7*** $\int_{t\;=\;0}^{t\;=\;tx}\frac{d}{dt}(SMcapbloodvol)\;=-Hepshuntflow+SMcapflow-PVflow$

**Table 1B.** Glucose equations *(number prefixes refer to positions in Figure 1)*.

$1\;IntestinalGlucoseabsorptionrate=(SGLT1Glucosepump*\frac{intestglc}{\left(intestglc+SGLT1Km)\right)}+Pgl*(intestinelumenglc-Smcapglc))$
$2\int_{t=0}^{t=tx}\frac{d}{dt}(SysVGlucose)=d/dt(SysVeinGlc)=+renalvglcflow+HVglcflow+somVglcflow–Venaglcflow+Hepshuntglcflow$
**4**A $\int_{t\;=\;0}^{t\;=\;tx}\frac{d}{dt}(hepaticglc)=d/dt(hepglc)=HAglcflow+PVglcflow+splenicVglcflow-HVglcflow-hepglcmetabolicrate$
**4** *B* $Hepaticglucosemetabolicrate=(V_{max}GLUT2*\frac{Glc_{Hep}}{K_{MGlut2}+GlcHe_{p}})*\frac{Ins_{\left(hep\right)}}{K_{ins}+Ins_{\left(hep\right)}}*Hepinsens*(1+GLP_{\left(hep\right)}Vmax*\frac{GLP}{K_{mGLP}+GLP_{\left(hep\right)}})$
$5\int_{t=0}^{t=tx}\frac{d}{dt}(Systemicglc)=d/dt(Systemicglc)=afferentsystemglucoseflow-systemicVglcflow-sysinsulin-depglucosemetabolicrate-peripheralinsulin-independentmetabolicrate.$
$6Renalurineglucosefl=(Renalglucoseflow*0.1–(TmSGLT2*\frac{Glc_{RenalA}}{Glc_{RenalA}+KmSGLT2})$
$7\int_{t=0}^{t=tx}\frac{d}{dt}(SMcapglc)=d/dt(afferentSMAglcflow+intestinalglcabsorptionrate-Portalveinglucoseflow-hepaticshuntglcflow)$
***Intestinal absorption,*** Intestinal absorption rate Glc pump is the glucose pump rate controlled by apical membrane SGLT1 V _max_; Glc _ (intest.Lum.)_ and Glc (SM cap) are the glucose concentrations, mM in the intestinal lumen and superior mesenteric capillary network, operational K _m_ is the affinity of SGLT1 activated pump is taken as 17 mM ^16, 17^ (Debnam & Levin, 1975 *b*). Intestinal glucose absorption rate = V _max_ _SGLT1_ * (Glc _(intest lumen)_/(Glc _(intest lumen)_ + K _m (SGLT1)_) + Pgl *(Glc _(intest lumen)_- Glc _(SM_cap)_). ***Net hepatic glucose uptake and hepatic glucose metabolism*** ***Hepatic glucose metabolic rate:-*** V _max_ _GLUT2_ is V _max_ of hepatic GLUT2/glucokinase, Glc _(Hep)_ the sinusoidal glucose concentration; K _m__GLUT2_ the K _m_ of hepatic GLUT2 for glucose mM; Ins _(Hep)_, the hepatic insulin concentration; Hepinsens, the hepatic sensitivity coefficient to insulin concentration; GLP-1_ _Hep_, the hepatic GLP-1concentration; GLN_ _Hep_, the hepatic glucagon concentration nM; Hep _GLNcoef_, the hepatic glucagon sensitivity. K _m_. _ins_, K _m_GLP-1_, K _mGLN_ are the K _m_s of insulin, GLP-1 and glucagon estimated to be within the range of known blood concentrations ^47, 48^ (Levy *et al.*, 1998; Wallace *et al.*, 2004).
$Hepaticglucosemetabolicrate=\left(\int hepglucosemetabolism=VmaxGLUT2*\frac{GlcHep}{KmGlut2+GlcHep}\right)*$ $\frac{HepIns}{Kmins+Ins}*Hepinsens*Insulinindependentmetabolicrate=insulininsensitivemetaboliccoef*$ $\frac{Glc_{sys}A}{\left(Glc_{sys}A+KmGLUT1\right)}*\left(1+GLPHep)*Vmax\frac{GLP}{KmGLP+GLPHep}\right)–HepGLNcoef*\left(\frac{HepGLN}{HepGLN+KmGLN}\right))$
***Renal glucose excretion.***
$Renalurineglucoseflow=(Renalglucoseflow*0.1–(TmSGLT2*\frac{Glc_{RenalA}}{Glc_{RenalA}+KmSGLT2})$
$Therenalfiltrationfractionis\mathrm{10}\%;SGLT2recoversrenaltubularglucoseK_{m}=0.1mMglucose.$

**Table 1C.** Insulin equations.

$2\;insulinsecretionrate=insulinglucosesensitivitycoef*\frac{Glc_{-sysA}}{\left(Glc_{{–sys}_{A}}+Km_{GLUT2}\right)}*\frac{GLP_{–SysA}}{\left(GLP_{–SysA}+KmGLP\right)}$
***4*** $\int_{t\;=\;0}^{t\;=\;tx}\frac{d}{dt}(Hepins)=d/dt(Hepinsulin)=-Hepinsulinloss–HVinsulinflow+HAinsulinflow+PVinsulinflowSplenic\&CeliacVinsulinflow$
***4*** *A* Hepinsulininactivationrate=Hep[insulin]×Hepinsulininactivationrate.
***5*** $\int_{t=0}^{t=tx}\frac{d}{dt}(sysins)=d/dt(sysinsulin)=-splenic\&celiacinsulinflow-somaticartinsulinflow-SMcapillaryinsulinflow+aorticinsulinflow-HAinsulinflow-RenAinsulinflow$
***5*** *A* Systemicinsulininactivationrate=[sysinsulin]*sysinsulininactivationrate.
***7*** $\int_{t=0}^{t=tx}\frac{d}{dt}(SMcapillaryinsulin)=d/dt(SMcapillaryinsulin)=-PVinsulinflow+Smcapillaryinsulinflow-Hepshuntinsulinflow+insulinsecretionrate.$

**Table 1D.** Incretin sub-model.

$GLP-1secretionrate=\frac{GLPGlc\_sens*Glc\_SMcap}{\left(Glc\_SMcap+KM\_GLUT2\right)}.$ Where GLP-1 glc __sens_ is the GLP-1 sensitivity to SM capillary glucose. Systemic GLP-1 degradation rate = GLP-1* GLP-1 _sysA_ loss coefficient. Splanchnic GLP-1 degradation rate = GLP-1 Hep loss rate*GLP-1_ _Hep_ *GLP-1_som is the somatic arterial GLP-1 concentration; GLP-1_Hep is the hepatic sinus GLP-1concentration*
***GLP-1 equations*** $1secretionrate=\frac{GLPGlc\_sens*Glc\_SMcap}{(Glc\_SMcap+KM\_GLUT2).}$ $2\int_{t=0}^{t=tx}\frac{d}{dt}(SysVGLP1)=d/dt(SysVGLP-1)=HVGLP-1flow+renalVGLP-1flow+GLP-1shuntflow+SomaticVGLP-1$ $4\int_{t=0}^{t=tx}\frac{d}{dt}(HepGLP1)=d/dt(HepGLP-1)=HAGLP-1flow+PVGLP-1flow-HVGLP-1flow+SP\&CEGLP-1flow-HepGLP-1loss$ $5\int_{t=0}^{t=tx}\frac{d}{dt}(SysAGLP1)=d/dt(SysAGLP-1)=aorticGLP-1flow-somaticGLP-1flow-SP\&CEGLP-1flow-SMAGLP-1flow-RenGLP-1flow-HAGLP-1flow.$

**Table 1E.** Glucagon Flow sub-model. Number prefixes refer to position in Figure 1.

Glucagon release from pancreatic islet α-cells is suppressed by systemic arterial glucose as follows:-
$Glucagonsecretionrate=GLN\_glc\_sensitivitycoef*(1–\frac{Glc\_sysA^{n}}{Glc\_sysA^{n}+\;Km\_GLUT1^{n}}).$
*The exponent n is found to give a good fit with n= 1 or 2*
$4\int_{t=0}^{t=tx}\frac{d}{dt}(HepGLN)=d/dt(HepGLN)=+PVGLNflow-HVGLNflow+HAGLNflow+SPCeGLNflow-HepGLNloss$
$5\int_{t=0}^{t=tx}\frac{d}{dt}(SysGLN)=d/dt(SysGLN)=-SplenicandceliacvGLNflow–SomGLNflow–RenVGLNflow–HAGLNflow+aorticGLNflow-afferent.SMAGLNflow$
$7\int_{t=0}^{t=tx}\frac{d}{dt}(SMAGLN)=d/dt(SMAGLN)=GLNsecretionrate–GLNshuntflow–PVGLNflow+afferentSMAGLNflow$

**Table 2.** Model parameters.

Right Ventricular pump	290
Left Vent pump	850
**Resistances**	**Hg.s ml ^-1^. ml ^-1^**
Aortic R	0.04
Pulmonary Artery R	0.06
Muscle R	0.06
Renal A R	0.1
SM A R	1
Hep A R	0.2
Vena cava R	0.001
Somatic Vein R	0.01
Renal V R	0.01
Portal V R	0.005
Hep V R	0.001
Hep shunt R	0.4
**Distensibility**	**mm Hg ^-1^**
Left Vent compliance	1E-4
Somatic vein compliance	0.01
Aortic compliance	0.001
SM cap compliance	0.005
Hep sin compliance	0.005
Splenic & Coeliac compliance	0.05
Renal compliance	0.05
Sys compliance	0.1
H sinusoid compliance	0.005
**Glucose parameters.**	
Intestinal glucose pump V	5 mmole s ^-1^
Paracellular P _gl_	0.15 μm s ^^-1^^
GLUT2 V _max_	3 mmole s ^-1^
Renal Glucose T _m_	1.2 mmole s ^-1^
GLUT1 K _m_	1.3 mM
GLUT2 K _m_	20 mM
Somatic glucose metabolic coef.	0.5 mmole s ^-1^
Somatic insulin-dependent metabolic coef.	8.5 mmole s ^-1^
Non-insulin-dependent metabolic coef.	0.65 mmole s ^-1^
B cell K _m (glucose)_	17 mM
**Insulin parameters**	
K _m insulin_	200 pM
Insulin loss rate (Hepatic)	1.4 s ^-1^
Insulin sensitivity coef.	0.5
insulin loss rate (Somatic)	0.1 s ^-1^
**Glucagon parameters**	
Glucagon coef. Hepatic	150
Glucagon loss rate (Hepatic)	0.5 s ^-1^
Glucagon coeff. (somatic)	150
Glucagon loss rate (somatic)	0.5 s ^-1^
K _m glucagon_	2 nM
**GLP-1parameters**	
K _GLP-1_	1 nM
GLP-1 loss rate (somatic)	0.22 s ^-1^
GLP-1 hepatic) sensitivity coef.	2
V _m_ GLP-1	20 nmole s ^-1^
GLP-1(somatic) sensitivity coef.	2

All the simulations were generated using Berkeley Madonna version 9.0. ( http://www.berkeleymadonna.com), a modelling and analysis program that solves simultaneous non-linear differential equations. It runs on Microsoft Windows 7–10, Macintosh and Linux platforms. The computer simulations are done using the option solving stiff non-linear simultaneous differential equations using the Rosenbrock simulation method ^51^ with a step time of 100 µs and error tolerance of 1×10 ^-8^. Simulations usually extend for 1500 virtual seconds, normally outputted at 5 second intervals. The numerical data output tables were subsequently processed in Microsoft Excel 2013 for Windows 2013 and graphed using the build-in Chart facility. Further analysis was done using self-generated Excel Solver macros, and the Levenberg-Marquardt, L-M, least squares minimizing routines available with Synergy Software Kaleidagraph version 3.52, ( www.synergy.com). This conveniently includes error estimations of the derived parameters.

### Model description

Cardiac output at rest is set at approximately 5.5 L/min and mean aortic blood pressure at ≈ 105 mm Hg. The core model blood vessel resistances and compliances are adjusted to obtain appropriate normal human steady state flows and pressures. The compartmental volumes are determined by their compliances, C and the transluminal pressure. Their initial and steady state values are adjusted to match known human values. The most pertinent compartmental compliances are the superior mesenteric capillary (SM cap) and hepatic sinusoidal beds. The circulating blood volume is assumed to be a third of the extracellular volume into which glucose, insulin and glucagon are distributed rapidly.

***The main components of the model of glucose circulation and metabolism***. Intestinal absorption, is modelled as active and passive parallel transmission elements connecting the intestinal lumen with the submucosal capillary bed. Passive glucose flows depend on the glucose concentration gradient existing between the intestinal lumen and modal sub-mucosal glucose concentration and linked via the passive intestinal paracellular glucose permeability. The active component to intestinal uptake is assumed to be a saturable function of luminal concentration with constant Na ^+^ concentration = 140 mM ( Figure 1 (1), Table 1B equation 1).

***Net hepatic glucose uptake NHGU and hepatic glucose metabolism***. Glucose flows via the portal circulation into the liver, where it is absorbed via sinusoidal GLUT2 and metabolized by insulin and GLP-1-dependent processes, the non-absorbed glucose flows via to the hepatic vein to the systemic circulation. The rate of hepatic glucose uptake and metabolism is controlled by the synergistic incretin and insulin dependent Vmax of hepatic GLUT2 and are tightly coupled to glucokinase activity, Glucose can also be regenerated by glucagon-dependent gluconeogenesis and glycogenolysis ( Figure 1 (4), Table 1B equation 4).

### Systemic glucose metabolism

Glucose enters into the systemic circulation via the hepatic vein ( Figure 1 (2), Table 1B equation 2). It is metabolized by either insulin-dependent processes in muscle and adipose tissue, to which is entry is controlled by the insulin- and GLP-1-dependent Vmax of GLUT4 (Km 2.5 mM glucose), Figure 1 (5), Table 1B equation 5. Additionally, insulin-independent glucose uptake processes in brain, bone and skin consume glucose, entry to these tissues is controlled via GLUT1 parameters (V _max_ and K _m_) Figure 1 (6), Table 1B equation 6.

### Insulin flow sub-model

Insulin is released by pancreatic islet β cells into the superior mesenteric blood compartment, partially in response to a Michaelis-Menten function of systemic arterial glucose concentration. GLUT2 is a rate determining step of this process ( Figure 1, Table 1C equation 1).

### Glucagon flow sub-model

Glucagon, like insulin, is released from the pancreatic islets (α-cells) into the superior mesenteric blood compartment and circulates in the splanchnic and systemic circulations its release is suppressed by raised systemic glucose as a hyperbolic function of glucose concentrations Ki controlled by GLUT2 ( Figure 1, Table 1E equation 1).

### GLP-1 sub-model

In contrast with glucagon and insulin, which are sensitive to systemic arterial glucose, incretin secretion rates are controlled by the splanchnic capillary glucose concentration. Incretins (GLP-1 and GLP-1-2) are released from proximal intestinal enteroendocrine L cells and flow directly into the portal blood compartment, the stimulus for their release is assumed to be the glucose concentration within this superior mesenteric capillary compartment, determined by GLUT2 K _m_ ( Table 1E equation 2).

***Estimation of the sensitivities of the flow and concentration variables***. Altering single parameters e.g. intestinal paracellular glucose permeability, P _gl_, or the glucose sensitivity of enteroendocrine cell GLP-1secretion have many important quantitative and qualitative effects on the flows of blood glucose hormones and incretins. These responses may be linear, where it is simple to estimate the sensitivity by linear regression, or hyperbolic. In this latter case the function is normally fitted to a hyperbolic curve, defined by two parameters, the maximal rate, V _max_, or the concentration of e.g. GLP-1, or the resistance to blood flow giving half maximal concentration, K _½_ or flow rates. These parameters are estimated by non-linear least squares fits of the hyperbolic function to the observed data. The standard error of these fits is < 5% and as it does not represent an experimental error is omitted. As there is significant interaction between several key effectors, e.g. GLP-1secretion rate and paracellular glucose permeability, P _gl_, a measure of this interaction is required. All of the 3D surface plots of the dependent variable, *z* with respect to alterations in the independent variables *x* and *y* can be fitted using least square regression or minimal Chi ^2^ fits either to the second order surface equation, where *z = a.x ^2^ + b.y ^2^ + c.x.y + d.x + e.y + f* or the equivalent third order equation.

The key coefficient required to estimate the degree of second order interaction between the two variables *x* and *y* is *c*. For ** positive x _*_y interactions *c* > 0 for negative x _*_y interactions *c* < 0. Examples of positive interaction are seen in Figure 5A, where SM arterial flow varies as an increasing function of both GLP-1secretion and paracellular glucose permeability, P _gl_. However, with P _gl_ = 0 or GLP-1≅ 0, SM flow is small 200 ml min ^-1^. SMA flow after feeding increases as a linear function of GLP-1 and as a hyperbolic function of P _gl_; K _½_ = 0.02 μm s ^-1^ and the interaction coefficient *c* for Figure 5D = 4.1, indicating a strong positive interaction between P _gl_ and GLP-1secretion, as can be seen from the upward elevation of the surface towards higher values of both independent variables. In contrast, during fasting, when intestinal glucose absorption is absent, although SMA increases with GLP-1secretion, there is no effect of altering P _gl_, so coefficient *c* = 0. Where the independent variables both independently *x* and *y* cause a reduction in response, i.e. negative response, as is the effect of increasing GLP-1secretion on SM capillary glucose during feeding, then when both are increased, *c* = -4.58 during feeding, but during fasting the response *c* = 0.

***Blood flow***. The simulations are simplified by assuming that superior mesenteric artery supplying blood to the small intestine is the only flow resistance directly responsive to glucose ( Table 1A equation 7, Figure 1 (7)).

All other blood flow changes are indirect reactions to this primary response. Blood flows are determined directly by the pressure gradients ΔP between the neighbouring nodal points in the circulation model ( Table 1A equation 7, Figure 1 (7)).

**Figure 2.**
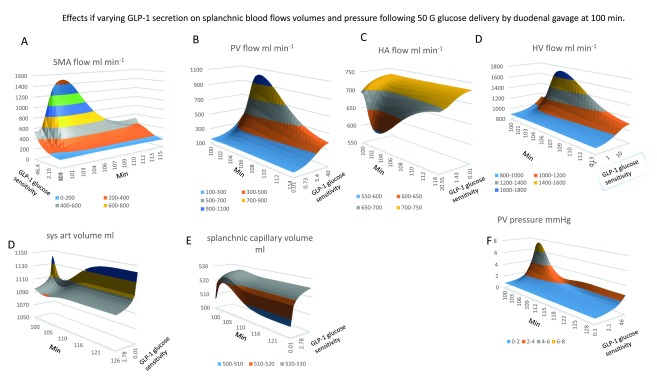
Effects of varying the glucose sensitivity of GLP-1 secretion on splanchnic blood flows, volumes and pressure following 50 G glucose delivery by duodenal gavage at 100min. All the graphs are contoured surface plots in which the *x* axis is the time coordinate, the *y a*xis is the GLP-1 sensitivity to glucose – this generates GLP-1at a rate proportional to the sensitivity and splanchnic blood glucose concentration, (Figure 1B GLP-1equations 1). With low GLP-1secretion the changes in glucose sensitive blood flow are reduced. **Panel A** The effects of GLP-1sensitivity and time on SMA flow. There GLP-1dependent increase in SMA flow response to glucose gavage peaks 3–6 min after glucose gavage and is sustained for 15–20min ( *K _½GLP-1 sens._ = 12; maximal flow rate 1500 ml min ^-1^; maximal flow rate 1500 ml min ^-1^*). **Panel B** Portal venous flow ml min ^-1^ versus GLP-1sensitivity and time. The graph has a similar GLP-1sensitivity and time course to SMA in ( **Panel A**). PV flow rises hyperbolically with GLP-1sensitivity *K _½GLP-1 sens._ = 12; maximal flow rate 1100 ml min ^-1^.* **Panel C**, the effects of GLP-1sensitivity and time after gavage on hepatic artery HA flow. The high GLP-1sensitivity is shown at front of the *y* scale. HA flows fall simultaneously with the rise in PV flow. This is due to the decreased aortic pressure and volume ( **Panel D**) resulting from the enlargement of the splanchnic volume ( **Panel E**). **Panel F** Effects of glucose sensitivity GLP-1secretion on portal venous PV pressure changes after glucose gavage. The rise in pressure mirrors the changes in PV flow ( **Panel B**) and SMA flow ( **Panel A**), ( *peak PV pressure is approximately 8mm Hg; K _½GLP-1 sens._ = 15*).

As blood flows and pressures within the network obey Kirchoff’s laws, flow changes in other parts of network result from passive reactivity. The initial and steady state compartment volumes are adjusted to match known human values. For typical compartmental pressure change generated by change in volume see Figure 2D and 2E and Table 2. Changes in compartmental volumes (ml) following perturbations in blood flow are determined by their compliances, C and changes in transluminal pressure, generated by the blood flows.

All other compartments in Figure 1 depend on their assigned initial volumes and compliances and the integrated inflows and outflows. The most relevant compartmental compliances are those determining the splanchnic blood volumes, i.e. the superior mesenteric capillary bed and the hepatic sinusoidal bed resulting from glucose-dependent alteration of SMA flow.

The total circulating blood volume is assumed to be a third of the extracellular volume into which glucose, insulin and glucagon are rapidly distributed in all accessible compartments ^52^. It is assumed that all the circulating glucose, hormones and incretin concentrations rapidly equilibrate between the circulating blood and their neighbouring extracellular fluid compartments. Thus the total circulating blood volume is 5 L and the fluid volume is initially and remains at approximately 15 L.

***Glucose flow sub-model***. Both splanchnic and systemic glucose circulations are incorporated within the core blood circulatory model. Ingested fluid entry and exit from the stomach, intestine and colon are programmed in order to fully replicate oral glucose tolerance tests. However, only a standard glucose dose via duodenal gavage delivery is shown in this present study. The key equations determining glucose flows are outlined in Figure 1. The parameters determining the rates of glucose flow and metabolism are shown in the Table 2.

***Explanation of the model components of glucose circulation and metabolism***. *Intestinal absorption*, is modelled by parallel active and passive transmission elements connecting the intestinal lumen with the submucosal capillary bed ( Table 1B Glucose equation 1).

Passive glucose flows depend on the glucose concentration gradient existing between the intestinal lumen and sub-mucosal capillary glucose concentration and the passive intestinal paracellular glucose permeability, P _gl_. The active component to intestinal uptake is assumed to be a saturable function of luminal concentration with constant Na ^+^ concentration = 140 mM. In addition to glucose entry via the superior mesenteric capillary bed, glucose also enters the splanchnic circulation via the superior and inferior mesenteric arteries, splenic and coeliac arteries ( Table 1B Glucose equations 2–7). Glucose concentrations, mM within each body compartment are obtained from the amounts of glucose (mmoles)/volumes (L) within each compartment.

***Net hepatic glucose uptake, NHGU and hepatic glucose metabolism (Glucose equation 4B)***. Glucose flows via the portal vein, PV into the liver, where it is absorbed via sinusoidal GLUT2 and metabolized by insulin and GLP-1-dependent processes starting with the enzyme glucokinase, the remaining non-absorbed glucose flows onwards via the hepatic vein, HV to the systemic circulation ( Table 1B Glucose equation 4A). The rates of hepatic glucose uptake and metabolism are controlled by the synergistic incretin and insulin-dependent V _max_ of hepatic GLUT2/glucokinase complex ( Table 1B Glucose equation 4A). It is assumed that GLUT2 and glucokinase activities are tightly coupled, so hepatic glucose metabolism is synergistically controlled by activation of coupled insulin and GLP-1 receptor ^53, 54^ that modulates the combined GLUT2- glucokinase V _max_. Glucose can also be added to the hepatic sinusoidal circulation by glucagon-dependent gluconeogenesis and glycogenolysis, ultimately rate-limited by hepatic glucose 6 phosphatase activity ^55^.

***Systemic glucose metabolism***. Glucose enters the systemic circulation via the hepatic vein (HV). It is consumed by insulin-dependent processes in muscle and adipose tissue, entry to which is controlled by the insulin- and GLP-1-dependent V _max_ of the glucose transporter GLUT4 ( Table 1B Glucose equation 5B).

Additionally, insulin-independent glucose uptake processes in brain, bone and skin consume glucose, entry to these tissues is controlled via GLUT1 parameters (V _max_ and K _m_) ^56, 57^ ** ( Table 1B Glucose equation 5A).

***Renal glucose excretion***. When the renal artery glucose concentration exceeds the ceiling for renal glucose reabsorption, glucose is excreted in urine at a rate proportional to the difference between renal glucose filtration rate (approximately 10% of renal artery flow and renal glucose re-absorptive capacity ( Table 1B Glucose equation 6). Urinary glucose loss does not significantly affect glucose metabolism in any of the simulations.

***Insulin flow sub-model***. Insulin is released from pancreatic islet β cells into the superior mesenteric blood compartment, partially in response to a GLUT2 Michaelis-Menten function of systemic arterial glucose concentration ( Table 1C Insulin equation 2 *,* Figure 10A). Glucose uptake via GLUT2 is the rate determining step of this process. However this rate is modulated by a glucose sensitivity coefficient, which is a function of systemic GLP-1 concentration, ^58, 59^. Like glucose, insulin circulates to the liver via the splanchnic circulation, but is partially inactivated within liver before passing to the systemic circulation, where it is also partially degraded ^60^ ( Table 1C Insulin equations 4A and 5A).

Insulin secretion rates are adjusted to give concentrations within the systemic circulation, similar to known concentrations in normal and T2DM states ( Table 2). The rates of insulin inactivation/degradation correspond with the reported inactivation rates t _½_ ≈ 2–3 min ^33, 48^) and adjusted to give a ratio of SMA insulin/peripheral venous insulin ≈ 2.0 ^61^.

***Glucagon flow sub-model***. Glucagon, like insulin, is released from the pancreatic islets (α-cells) into the superior mesenteric blood compartment and circulates in the splanchnic and systemic circulations. Glucagon release responds as an inverse hyperbolic function of the systemic glucose concentration and is regulated only with a glucose-sensitive coefficient (Figure 1C, Table 1E Glucagon equation 2, Figure 10C).

On contact with hepatocytes glucagon stimulates hepatic glucose production by gluconeogenesis and glycogenolysis ( Table 1B Glucose equation 4B). These processes result in net glucose release, into the systemic circulation. Glucagon, like insulin, decays within the circulation with a similar degradation half-time of 2–3 min, but is more slowly degraded by liver than insulin, so that the portal to arterial glucagon ratio is reported to 1.2–1.4 ^61^. For present purposes the liver is assumed to be a limitless source, of gluconeogenesis from either glycogen or from fat and protein stores. This condition obviously applies only to the short term (1–2 days).

***Incretin sub-model***. Incretin secretion rates are controlled by the splanchnic capillary glucose concentration and like insulin and glucagon, incretins flow directly into the portal blood compartment *(* Table 1D GLP-1 equation 1 *).* The stimulus for GLP-1 release is dependent on glucose concentration within this superior mesenteric capillary SM cap compartment, determined by GLUT2 K _m_^35, 62– 64^. Thus incretin release from enteroendocrine L cells differs from glucagon and insulin release from pancreatic islets; these are sensitive to systemic arterial glucose; whereas GLP-1 release is activated by splanchnic glucose concentrations. Like insulin and glucagon, GLP-1 has a half-time of degradation of 2–3 min; this is modelled by Table 1D GLP-1 equation 2, and Figure 10C.

**Figure 3.**
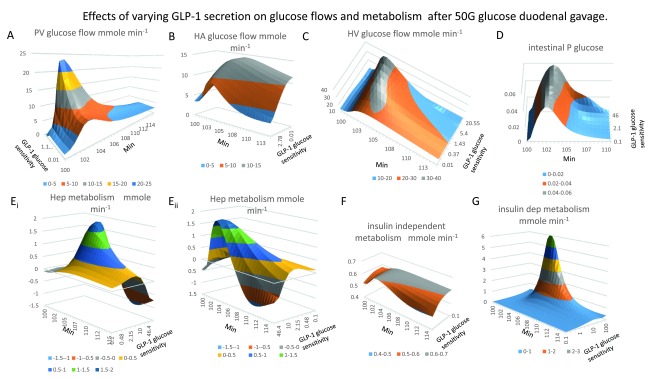
Effects of varying glucose sensitivity of GLP-1secretion on glucose flows and metabolism following 50 G glucose duodenal gavage at 100min. **Panel A** The rise and fall of PV glucose flow following gavage, ( *peak flow rate 18 mmole min ^-1^ at 2–5 min after gavage, K _½GLP-1 sens._ = 12*). **Panel B**, HA glucose flow does not fully reciprocate PV glucose flow since raised GLP-1only reduces HA glucose to a small extent ( *14.3 to 7–8 mmole min ^-1^ between 4–6 min after gavage).* **Panel C**, HV glucose flow is the sum of PV and HA flows shown in ( **Panels A** and **B**). **Panel D** The rise off unidirectional intestinal glucose permeability following gavage. Intestinal glucose permeability varies transiently with the transluminal glucose concentration gradient. This rises with the increase in luminal glucose concentration and falls from the peak when glucose gavage ceases and luminal glucose concentration falls and splanchnic capillary glucose concentration rises due to glucose absorption (see Figure 4 **Panel G**) Raising GLP-1glucose sensitivity increases the peak glucose permeability by 20%. GLP-1increases peak flow glucose permeability from the baseline at the start of gavage compared with maximal glucose gradients by five-fold. **Panels Ei**, **Eii** Mirror views of the effects of GLP-1on hepatic glucose metabolism following gavage. Negative values signify negative net glucose uptake NHGU i.e. positive glucose outflow resulting from glucagon stimulation and suppression of GLP-1and insulin signalling to liver. High GLP-11 sensitivities increase NHGU during times of peak PV glucose flow, however later times, high GLP-1sensitivities leads indirectly to very high rates of glucagon-dependent gluconeogenesis *K _½GLP-1 sens._ = 18*. **Panel F** Peripheral glucose metabolism increases only slightly with raised systemic glucose concentration following glucose absorption and falls when high rates of glucose sensitive GLP-1secretion drive metabolism to induce hypoglycaemia. **Panel G** Insulin-dependent glucose metabolism is extremely sensitive to glucose sensitive GLP-1secretion. The maximal rate is > 100-fold higher than fasting rates. With low GLP-1 net hepatic glucose output is reduced and hepatic uptake reduced during the absorption phase 100–145 min. Low GLP-11 secretion reduces peripheral insulin sensitive glucose uptake.

**Figure 4.**
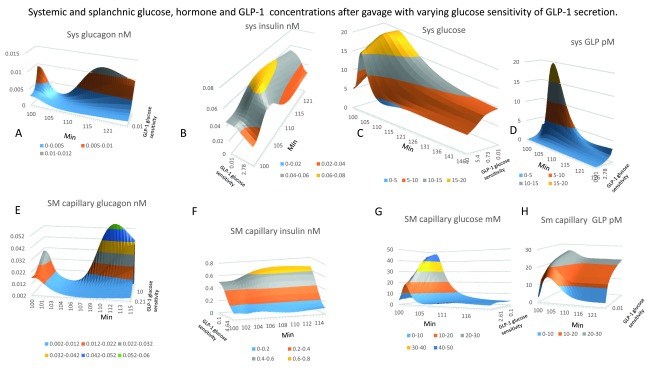
Effects of varying the GLP-1glucose sensitivity of secretion on systemic and splanchnic glucose, hormone and GLP-1concentrations post duodenal glucose gavage. **Panel A–D** systemic concentrations of glucagon, insulin, glucose and GLP-1. **Panels E–H** splanchnic concentrations of glucagon, insulin, glucose and GLP-1respectively. Note that the splanchnic concentrations are generally nearly twice those in the systemic circulation. Peak SM capillary glucose concentration decreases as GLP-1 secretion rate increases, ( *GLP-1glucose sensitivity range 0–50 K _½GLP-1_=7.2 required to give half maximal splanchnic glucose concentration maximum splanchnic glucose ranging from 45 to 22 mM as GLP-1is increased).* After the glucose absorptive phase glucagon levels rapidly recover with high rates of GLP-1secretion in both splanchnic blood (K _½ GLP-1sec_ = 10) and in systemic blood (K _½ GLP-1sec_ = 5). With high rates of GLP-1-1 secretion glucagon remains high in both splanchnic and systemic blood until fasting is relieved. **Panel B** and **F** Insulin concentration in SM-cap is 2.5-fold higher than in systemic blood. Splanchnic insulin is nearly 10x higher with low GLP-1 than with high rates of GLP-1secretion. **Panels C** and **G** Splanchnic glucose exceeds systemic glucose by 1-5-2 fold during glucose absorption but falls below that of systemic glucose particularly with high rates of GLP-1secretion during fasting and in the later post absorptive phases of digestion. *Fasting glucose in systemic blood with high GLP-1 glucose 5.6 mM; with low GLP-1, glucose 9.6mM; splanchnic blood glucose with high GLP-14.4 mM and with low GLP-1 -1 glucose 3.6 mM. In contrast during the absorptive phage splanchnic glucose with low rates of GLP-1secretion glucose 47.5 mM exceeds systemic glucose 19.5 mM this is caused by the lower rates of SMA flow than with higher rates of GLP-1secretion.* **Panels D and H** Splanchnic GLP-1always exceeds systemic glucose however with high rates of GLP-1secretion due to high glucose sensitivity, *the peak splanchnic GLP-126 pM observed during glucose absorption is similar to systemic 19.7 pm whereas during fasting systemic GLP-12–3pm and splanchnic GLP-1 20–22 pM.*

 In Figure 2– Figure 4 the effects of altering the glucose sensitivity over a range from (0.1–100) of GLP-1 release are shown on the key pressure, volume, flow and concentration variables affecting glucose distribution and metabolism, as functions of time after initiation of duodenal glucose gavage at 100min. Increasing glucose sensitivity over the range (0.1–100) increases the GLP-1 concentration in both splanchnic and systemic circulation by around 20 fold, ( Figure 3D and 3H) *(The linear regression coefficient of splanchnic capillary GLP-1 concentration with GLP-1-glucose sensitivity coefficient is 0.58 ± 0.01 and for systemic arterial GLP-1, the coefficient is 0.5 ± 0.007).*

The effects of a standard oral glucose tolerance test, OGTT of 50 G glucose delivered by duodenal gavage over a period of twenty minutes are used in all simulations to demonstrate the comparative effects of these altered conditions on glucose flows and metabolism.

The effects of two major physiological variables, the GLP-1 sensitivity to glucose and the paracellular glucose permeability, P _gl_ on glucose absorption and its distribution and metabolism are displayed in the first part of this paper. Glucose sensitivity of GLP-1 release is the main regulator of superior mesenteric arterial response to glucose and the second variable P _gl_ affects the passive paracellular rate of glucose flow and hence its sensitivity to splanchnic capillary flow rates. The other major effects of altered GLP-1 secretion rates will be described in the first part of the Results section.

In the second part of this paper variations of two parameters, hepatic pre-sinus resistance and portosystemic shunt resistance, PSS R associated with NAFLD and NASH on hormonal and incretin changes affecting glucose absorption and metabolism will be examined.

No other parameters, or coefficients are altered during these simulations. All the other parameters used are the same as in Table 2.

Most of the graphs shown are 3D representations in which the arrays of dependent variable *z* are plotted versus array vectors of *x* ( *time*) and *y* {independent variables, ( *resistances, permeabilities, etc.*)}. This method of variable mapping using 3D surface graphs with Excel Chart facilities demonstrates the non-linear interactions between variables, however, only the time axis and the dependent variable are an exact linear or logarithmic maps of the independent variable The K _½_ and “c” estimates of *x, y* interactions are all obtained with exact fits.

## Results

Raw data for ‘A computer model simulating human glucose absorption and metabolism in health and metabolic disease states’ The source data for each figure is included. Click here for additional data file. Copyright: © 2016 Naftalin RJ 2016 Data associated with the article are available under the terms of the Creative Commons Zero "No rights reserved" data waiver (CC0 1.0 Public domain dedication).

**Figure 5.**
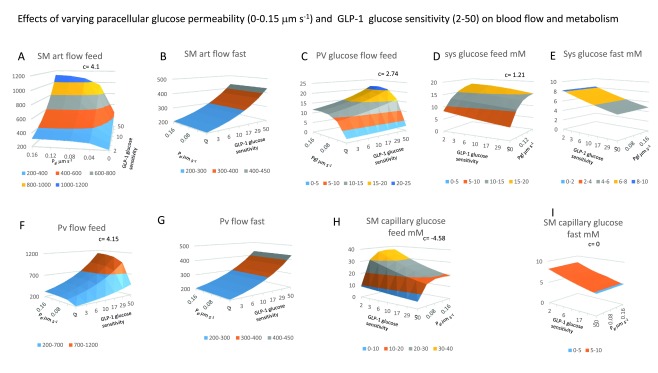
Effects of varying paracellular glucose permeability P _gl_ (0–0.15 μm s ^-1^) on blood flow and metabolism. All the panels show 3D surface plots of the effects of two variables GLP-1glucose sensitivity (2–50) that controls GLP-1secretion rate with changes in splanchnic glucose concentration ( *GLP-1equations 1*) and intestinal paracellular glucose permeability, P _gl_ (0–0.15 μm s ^-1^). The interaction between these variables is shown as the coefficient *c*. All the panels show paired effects contrasting the interactions at peak splanchnic glucose flow (feed) with those during when splanchnic glucose is at a minimal value ( *fast* **panels D** and **E**, the feeding and fasting values are determined at maximal and minimal systemic glucose concentrations. **Panel 5A** SMA flow after feeding increases as a linear function of GLP-1and as a hyperbolic function of P _gl_; K _½max_ = 0.02 μm s ^-1^ and the interaction coefficient *c* for = 4.1, indicating a strong positive interaction between P _gl_ and GLP-1secretion, as can be seen from the upward elevation of the surface towards higher values of both independent variables. **Panel 5B** During fasting, in contrast to effects seen with feeding in **panel 5A** there is no effect of altering P _gl_ although SMA increases with GLP-1secretion, ( *coefficient c* = 0) when intestinal glucose absorption is absent. **Panel 5C** There is a synergistic response of portal blood flow and glucose flow as a result of the interaction between P _gl_ and GLP-1secretion which leads to both increased splanchnic blood flow and glucose concentrations *c*= 2.74. With P _gl_ intestinal paracellular permeability = zero, increased SMA in response to raised GLP-1is almost without effect on portal glucose flow rates. Increasing P _gl_ from 0 to 0.16 μm s ^-1^ with a constant rate of GLP-1secretion (= 50) and low pre-sinusoidal resistance (0.005 mm Hg.s ml ^-1^), results in a hyperbolic increase in portal venous glucose flow from a base of 2.45 mmole min ^-1^ to a maximal flow of 22.3 *±* 1.37 mmole min ^-1^, the P _gl_ giving half maximal increase in glucose flow is 0.024 *±* 0.007 µm s ^-1^. **Panels F** and **G** As with SMA flow see **Panels A** and **B** portal vein flows increase synergistically with increases in GLP-1and P _gl_ during when glucose is present in the splanchnic circulation c= 4.15, but during fasting Pgl effects are absent *c*= 0. **Figure 5H** There is a relatively high degree of negative interaction between the rate of GLP-1secretion and P _gl_ on splanchnic capillary glucose concentration, c= -4.58 due to both dilution of the intestinal glucose absorbate by the higher capillary blood flow rate, however as already shown glucose flow rate there is a positive interaction between P _gl_ with GLP-1-1 on PV glucose flow rates c = 1.21. When glucose paracellular permeability is high there the glucose uptake from intestine to the splanchnic blood is increased by high rates of capillary flow induced by GLP-1secretion. This due to the raised glucose gradient between the intestinal lumen and the submucosal capillaries.

**Figure 6.**
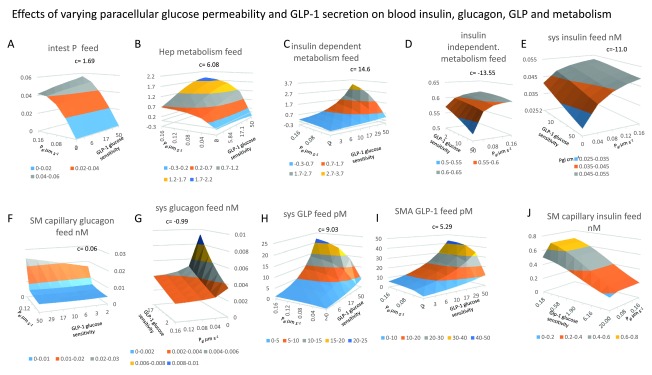
Effects of varying paracellular glucose permeability and GLP-1secretion on metabolism. **Panel A**, Unidirectional intestinal permeability decreases as SM capillary glucose concentrations increase ( Figure 3D). Unidirectional glucose permeability increases as a hyperbolic function of increasing paracellular permeability P _gl_ ( *K _½_ = 0.045 μm s ^-1^ and GLP-1glucose sensitivity K _½_ = 5.5, c = 1.69*). The positive interaction between paracellular permeability and glucose sensitive SMA flow indicates that raising capillary flow increases unidirectional permeability only when the paracellular leakage is fast enough to increase splanchnic capillary glucose concentration enough to retard permeability substantially if not cleared by splanchnic blood flow. ( **Panels 6B, 6C** and **6D**). During feeding increased rates of GLP-1secretion and intestinal glucose permeability P _gl_ synergistically increase NHGU ( *c* = 6.08), and peripheral glucose metabolism ( *c* =14.6). ( **Figure 6D**), Insulin-independent metabolism (c= -13.55) decreases from the more intense competition for systemic glucose from insulin dependent tissues. ( **Panel 6E**) Systemic insulin and ( **Panel 6H**) GLP-1concentrations also increase with increasing P _gl_ (K _½_ ≈ 0.03 µm s ^-1^).

**Figure 7.**
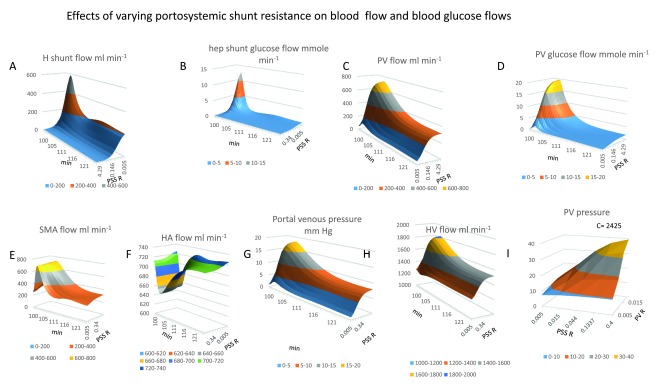
Effects of varying portosystemic shunt resistance on blood flow and glucose flow. **Panel 7A** Hepatic shunt blood flow increases as PSS resistance diminishes giving half maximal portal venous glucose flow, ( *coefficients V = 600 ml min ^-1^; K _½_= 0.025 mm Hg.s ml ^-1^ is maximum 3–4 min after gavage).* A slower but more prolonged rise occurs 20–30 min after gavage. **Panel 7B** Portosystemic glucose flow 3–4 min after glucose gavage. Glucose flow increases with decreasing PSS resistance ( *K _½_ = 0.05 mm Hg.s ml ^-1^*). **Panel 7C** PV flow decreases from its peak at a slower rate t _½_ ≈ 7.5 min to reach a plateau phase. During this plateau phase PV flow also decreases as a hyperbolic function of PSS resistance ( *K _½_ = 0.028 Hg.s ml ^-1^)* **Panel 7D** With zero PSS flow PV glucose flow has peak of approximately 20 mmole min ^-1^ PV glucose flow decreases (t _½_ = 1.2 min, with zero shunt flow and t _½_ = 0.45 min with high shunt flows). **Panels 7E** and **7F** PSS resistance change has negligible effects on either SM arterial blood flow or HA blood flow. **Panel 7G** Increasing PSS decreases peak portal venous pressure *(K _½_ = 0.05 Hg.s ml ^-1^ occurs at 5-5 min after the beginning of gavage, the t _½_ = 5–6 min of peak portal pressure decline*). **Panel 7H** HV flow is maximal during peak glucose absorption 1500–1800 ml min ^-1^ 5 min after the start of gavage. HV flow decreases as a hyperbolic function of PSS resistance *(K _½_ = 0.03 Hg.s ml ^-1^).* **Panel 7I** There is a strong interaction between PSS and presinusoidal resistance on hepatic shunt flow *(c = 2425)*; when GLP-1secretion rates are high reducing the PSS resistance below 0.027 Hg.s ml ^-1^ reduces peak PV pressure by 50%.

**Figure 8.**
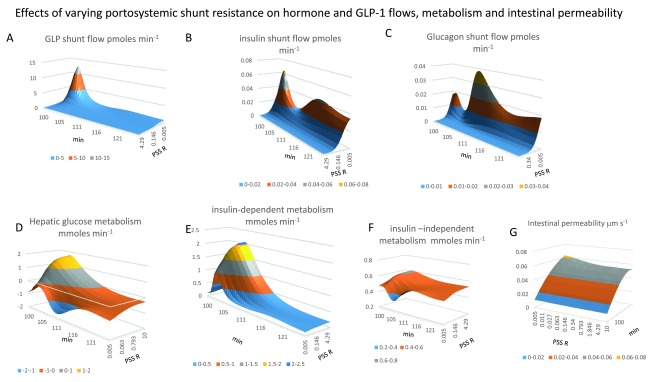
Portosystemic shunting effects on insulin, glucagon and GLP-1shunt flows and metabolism. **Panel 8A** GLP-1flow increase as hyperbolic function of PSS ( *the shunt resistance giving half maximal GLP-1flow is 0.027 Hg. s ml ^-1^, Peak flow 3 mins after the start of duodenal glucose gavage and decreases very rapidly (t _½_ ≈ 3 min)*. **Panel 8B** Insulin flow via the PSS peaks 2.5–3 min following the start of glucose gavage. *The shunt resistance giving half maximal peak insulin flow (K _½_ = 0.063 Hg.s ml ^-1^). A second wave of insulin flow via the shunt is seen with low shunt resistance (K _½_ = 0.03 Hg.s ml ^-1^).* **Panel 8C** When shunt resistance is ≤ 0.015 Hg.s ml ^-1^ glucagon flows via the PSS in two waves, The first wave peaks ( *1–2 min after gavage, flow rate of 20 fmoles min ^-1^ and t _½_ = 1.5 min decrease*). The second glucagon wave ( *peaks at 38 fmoles min ^-1^, 8–10 min after gavage shunt resistance is K _½_ ≈ 0.055 Hg.s ml ^-1^ (decay t _½_ = 10–15min).* **Panel 8D** Opening the PSS resistance < 0.05 Hg.s ml ^-1^ curtails the effect of GLP-1on hepatic glucose metabolism. With high shunt flows of glucose gavage net hepatic glucose uptake, NHGU, switches 6 minutes after the start glucagon-activated gluconeogenesis. **Panel 8E** Both hepatic ( **panel 8D**) and peripheral insulin-dependent ( **Panel 8F**) glucose consumption peaks are reduced at high rates of GLP-1secretion and a large PSS ( *K _½_ = 0.02 Hg.s.ml ^-1^*). The peaks occur earlier and end sooner. **Panel 8F** Insulin independent metabolic rate is stable over a wide range of PSS but is decreased with open PSS resistance < 0.02 Hg.s ml ^-1^ simultaneously with the decrease in peripheral glucose concentration. **Panel 8G** Unidirectional intestinal glucose permeability increase after gavage as the glucose gradient between intestinal lumen and splanchnic capillaries increases with luminal glucose concentration, it also increases slightly 19% with increased PSS due to decreased splanchnic glucose concentration, ( Figure 10A).

**Figure 9.**
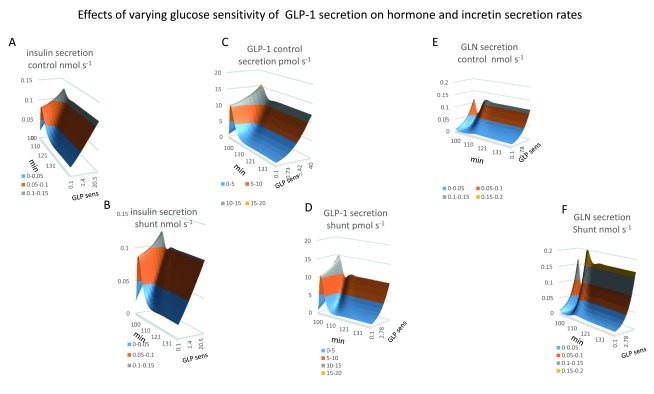
Effects of GLP-1secretion on the time course of insulin secretion. **Panel 9A**. Insulin secretion rates are increased during the glucose absorptive phase of metabolism. This increase is stimulated directly be systemic glucose concentration and by the glucose sensitivity of GLP-1secretion. During fasting insulin secretion rates are directly proportional to GLP-1glucose sensitivity however during peak glucose absorption insulin secretion rates vary to a much lesser extent as with low rates of GLP-1systemic glucose is raised and therefore compensates for lack of glucose sensitivity of GLP-1secretion. 1 ( *K _½GLP-1gluc sens_ = 0.80, V _max_= 0.41 nmol s ^-1^)* **Panel 9B Insulin secretion rates with PSS** ( *K _½GLP-1gluc sens_ = 0.72, Vmax0.375 nmol s ^-1^)* **Panel 9C GLP-1secretion is very similar to insulin**, GLP-1secretion increases rapidly during the glucose absorptive phase of metabolism and tails of splanchnic glucose is diminishes during the course of metabolism. During fasting GLP-1secretion is hyperbolically dependent on glucose as glucose sensitivity of GLP-1 *K _½_ = 4.4 Vmax 12.3 12 pmol s ^-1^)* secreting cells in splanchnic blood is concentrations are lower with high rates of GLP-1secretion ( Figure 4G). **Panel 9D** **GLP-1secretion with PSS** glucose sensitivity of GLP-1 *K _½_ = 4.6 Vmax10.3 pmol s ^-1^ Shunting reduces insulin secretion by approximately 20%.* **Panel 9F** **Shunting increases glucagon secretion rates**. The increase is a hyperbolic function of GLP-1 glucose sensitivity *(K _½_ = 6.7 Vmax = 0.12 nmol s ^-1^).* During glucose absorption glucagon secretion rates decrease as systemic glucose increases. The decrease is negligible with low rates of GLP-1secretion due to the slow rise in systemic glucose.

**Figure 10.**
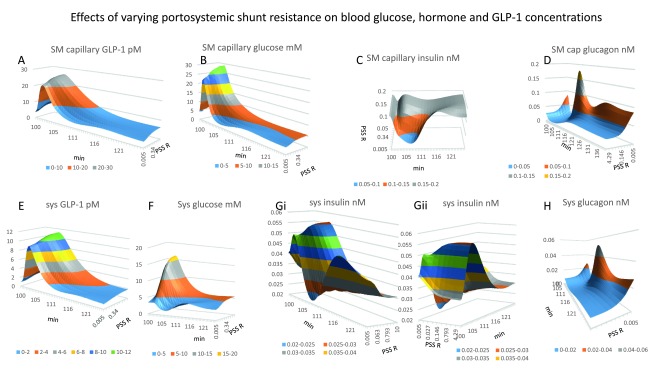
**Panels 10A and 10E** Portosystemic shunting has a relatively small effect on systemic and splanchnic GLP-1concentrations. **Figure 10 Panels 10A** and **10E** Portosystemic shunting has a relatively small effect on systemic and splanchnic GLP-1concentrations. **Panel 10B** and **10F** Peak systemic glucose decreases as PSS increases ( *PSS resistance K _½_= 0.05 Hg.s.ml ^-1^).* **Panels 10C** and **10G**, Splanchnic insulin is decreased by shunting 2–7 min after duodenal gavage *(PSS resistance K _½_= 0.145 Hg.s.ml ^-1^*). The decrease in splanchnic insulin coincides with a shunting-dependent increase in systemic and splanchnic glucagon ( **Panels 10D**, **10H**). Portosystemic shunts increase fasting systemic insulin concentrations *PSS resistance K _½_= 0.06 Hg.s.ml ^-1^)*. **Panels 10D** and **10H**. Systemic and splanchnic glucagon concentrations have the relatively the largest responses to portosystemic shunt opening. As well as an early peak at 10 min after gavage *(PSS resistance K _½_= 0.06 Hg.s.ml ^-1^),* a second later sustained rise in both systemic and splanchnic glucagon *(PSS resistance K _½_= 0.075 Hg.s.ml ^-1^).* **Panel 10F Fasting glucagon secretion rates with shunting increase hyperbolically with GLP-1glucose sensitivity** *K _½_ = 9.5 Vmax 0.19 nmol s ^-1^)*.

**Figure 11.**
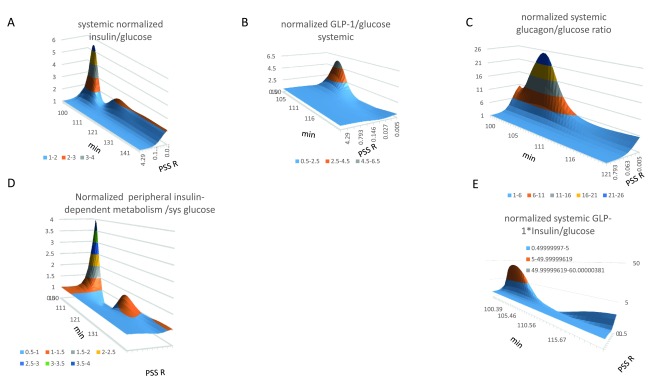
Effects of shunting on normalized systemic insulin/GLP-1-dependent metabolism/glucose ratios. **Panel 11A** Opening the PSS resistance from 40 to 0.005 mm Hg.s ml ^-1^, increases the normalized systemic insulin: glucose ratio to 2.1 in the fasting state *(K _½_ = 0.03 mm Hg.s ml ^-1^)* The normalized systemic insulin: glucose ratio increases as a hyperbolic function to maximum of 5.4 as shunt resistance falls ( *K _½_ = 0.03–0.04 mm Hg.s ml ^-1^).* Two peaks in the systemic insulin: glucose ratio (Figure 11A) The second smaller, longer lasting rise in the insulin/glucose ratio coincides with the second wave in hepatic gluconeogenesis/glucose ratio ( Figure 12E) ( *K _½_ = 0.06 mm Hg.s ml ^-1^*) and peripheral insulin-dependent metabolism ( *K _½_ = 0.015 mm Hg.s ml ^-1^*) (Figure 11D). **Panel 11B** Opening the PSS increases GLP-1/glucose ratio as a hyperbolic function of shunt opening ( *K _½_ = 0.015 mm Hg.s ml ^-1^*) the ratio peaks 5 min after gavage, and thereafter decreases (t _*½*_ = *2.5–3 min from the peak maximum).* **Panel 11C** Opening the PSS increases glucagon/glucose ratio as a hyperbolic function of shunt opening ( *K _½_ = 0.015 mm Hg.s ml ^-1^*) the ratio peaks 5.5 min after gavage, and thereafter decreases (t _*½*_ = *3 min after the peak maximum).* With a wide open shunt the glucagon/glucose ratio increases continuously during fasting owing to glucagon stimulated gluconeogenesis. **Panel 11D**, GLP-1 and insulin interactively stimulate systemic glucose metabolism in insulin-sensitive tissues. Plots of the product of the normalized GLP-1. Insulin product/glucose peak 4.5 min after gavage. Shunting raises the GLP-1.insulin product 30-fold increase above that without shunting. The enhancement remains during the later digestive periods. **Panel 11E** The normalized product of GLP-1*insulin in systemic blood increases as a hyperbolic function of PSS resistance. ( *K _½_ = 0.01 mm Hg.s ml ^-1^*to a *maximum 30-fold above the level with without shunting 7 min after gavage; t _½_ = 2.5–3 min from the peak maximum a residual increase remains throughout the later digestive phase. (K _½_= 0.08 mm Hg.s ml ^-1^).*

**Figure 12.**
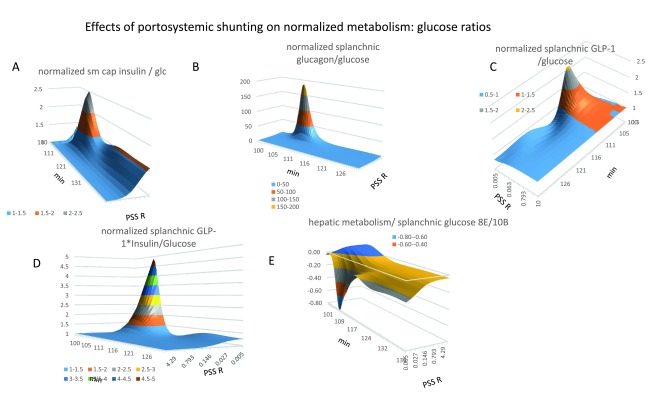
Effects of shunting on normalized splanchnic insulin/GLP-1-dependent metabolism/glucose ratios. **Panel 12A** Opening the PSS resistance from 40 to 0.005 mm Hg.s ml ^-1^, increases the normalized splanchnic insulin: glucose ratio in the fasting state to 2.1 *(K _½_ = 0.03 mm Hg.s ml ^-1^)* Two peaks in the splanchnic insulin/glucose ratio (Figure 12 **panel A**). The second smaller, longer lasting rise in the insulin/glucose ratio coincides with the second wave in hepatic gluconeogenesis/glucose ratio (Figure 12 **panel E**) ( *K _½_ = 0.06 mm Hg.s ml ^-1^*) and peripheral insulin-dependent metabolism ( *K _½_ = 0.015 mm Hg.s ml ^-1^*) ( Figure 11 panel D). **Panel 12B** Opening the PSS increases splanchnic glucagon/glucose ratio as a steep hyperbolic function of shunt opening ( *K _½_ = 0.01 mm Hg.s ml ^-1^*) the ratio peaks 8 min after gavage, and thereafter decreases (t _*½*_ = *2.5 min after the peak maximum).* **Panel 12C** Opening the PSS increases splanchnic GLP-1/glucose ratio as a hyperbolic function of shunt opening ( *K _½_ = 0.015 mm Hg.s ml ^-1^*) the ratio peaks 6 min after gavage, and thereafter decreases (t _*½*_ = *2.5–3 min from the peak maximum).* **Panel 12D** The normalized product of GLP-1*insulin in splanchnic blood increases as a hyperbolic function of PSS resistance. ****( *K _½_ = 0.05 mm Hg.s ml ^-1^*, *peak maximum is 7 min after gavage; t _½_ = 2.5–3 min from the peak maximum) The shunting dependent increase in splanchnic blood peaks approximately 5× higher and a residual increase remains throughout the later digestive phase. (K _½_= 0.08 mm Hg.s ml ^-1^)*. **Panel 12E** The ratio of hepatic metabolism/splanchnic glucose decreases falls dramatically during the early phase of glucose absorption when the PSS is opened (K _½_ = 0.015 mm Hg.s ml ^-1^) peaking 10 min after starting gavage.

**Figure 13.**
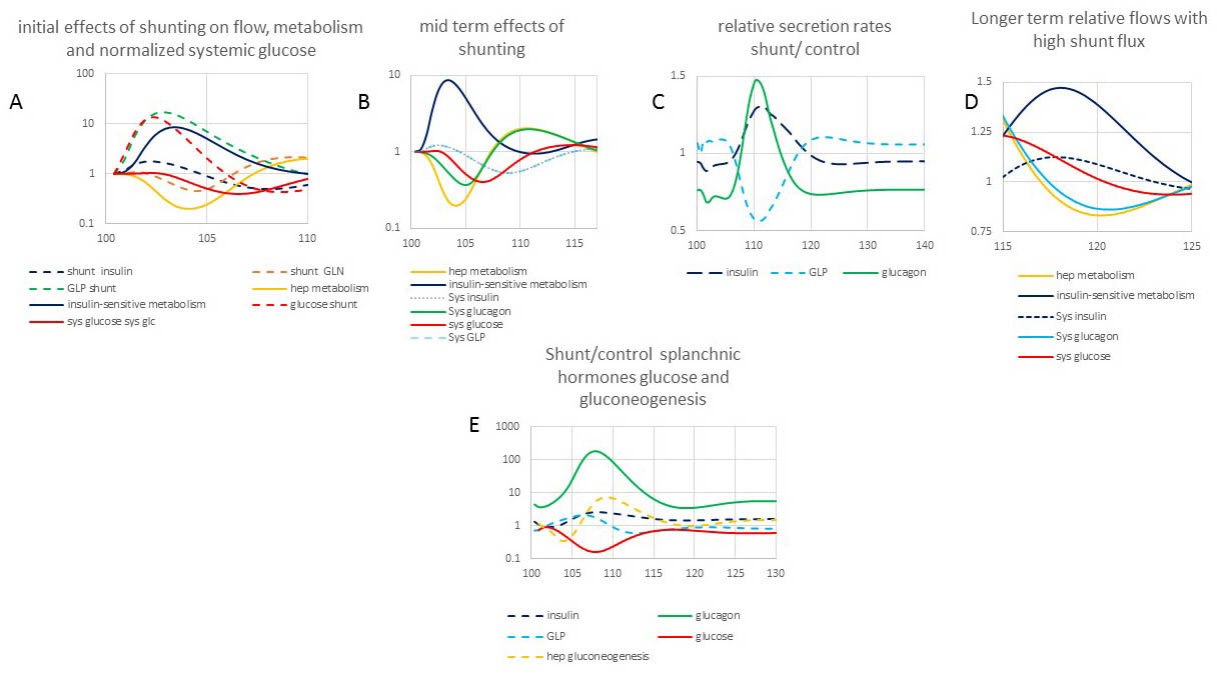
Panels 1–D The time courses of normalized shunt flow of glucose, insulin, glucagon, GLP-1 and peripheral insulin sensitive metabolism, hepatic metabolism. **Panel C** normalized shunt/control insulin, GLP-1 and glucagon secretion rates. Panel E Shunt/control ratio of glucose, insulin, GLP-1 and glucagon in splanchnic blood and hepatic gluconeogenesis rates (positive).

**Figure 14.**
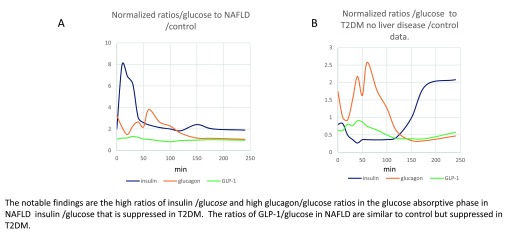
Ratios of insulin/glucose; GLP-1/glucose; glucagon/glucose disease/control (primary data from (Junker *et al.* 2016). **Panel A** Normalized ratios of systemic insulin/glucose; glucagon/glucose and GLP-1/glucose in patients with NAFLD. **Panel B** Normalized ratios insulin/glucose, glucagon/glucose and GLP-1/glucose in patients with T2DM having no liver disease/control data.

## Integration of intestinal glucose absorption with glucose metabolism

### Blood flow simulation

The initial aim was to model the interaction between incretin-induced reduction in SMA blood flow resistance and glucose absorption. Simulations of glucose-induced blood flow changes are shown in Figure 2. SMA ( Figure 2A) and portal blood flow ( Figure 2B) rise from a fasting rate of approximately 500 ml min ^-1^ to 1500 ml min ^-1^ during peak glucose absorption rates, similar to changes reported by 50. Hepatic arterial flow decreases simultaneously from 700-560 ml min ^-1^ ( Figure 2C). This mirrors the hepatic arterial buffer response ^65^, ascribed to a reflex action activated by intrahepatic release of adenosine by portal blood flow ^66^. However, here no humoral or nerve responses are programmed, so the reciprocal changes in HA flow with PV flow are due entirely to the direct mechanical compensatory changes resulting from application of Kirchhoff’s current law within the series-parallel circulatory network of blood vessels. Flow and volume changes resulting from the increases in portal venous flow and splanchnic blood volume, increase splanchnic volume ( Figure 2E), with consequential decreases in systemic arterial volume ( Figure 2D), blood pressure: aortic BP decreases from mean level of 110 mm Hg to around 90 mm Hg. Similar phenomena may account for the post-prandial hypotension frequently observed in elderly humans ^67^. The increase in SMA blood flow following release of incretins GLP-1 increases portal blood pressure from 1.5–7.5 mm Hg ( Figure 2F). The extent of this increase depends on a number of factors, as will be discussed. Raised portal venous pressure lasts as long as the splanchnic blood vessels are exposed to hyperglycaemia and SMA blood flow is raised ( Figure 2A).

***Glucose flows***. As both PV flow ( Figure 2B) and superior mesenteric capillary (SM cap) glucose concentrations increase ( Figure 4C) during the glucose absorptive phase, PV glucose flow rises by about ten-fold from 2.4–24 mmoles min ^-1^ ( Figure 3A). HA glucose flow increases only by threefold from 2 to 6 mmoles min ^-1^ ( Figure 3B). Consequently, during the intestinal absorptive phase, PV supplies 80% and HA 20% of hepatic glucose, whereas during fasting periods, hepatic glucose inflows from the PV and HA are nearly equal. During the early glucose absorptive phase HV glucose outflow only slightly exceeds PV glucose inflow, but in the later digestive phases HV glucose outflow greatly exceeds PV glucose inflow ( Figure 3C).

With normal high rates of GLP-1 secretion ( *systemic arterial GLP-110-20nM*; Figure 4D), splanchnic glucose concentration ( Figure 4G) rises transiently to 20mM, then subsides to 5 mM as the SMA blood flow and insulin, glucagon and GLP-1 regulate the systemic capillary glucose. Systemic arterial glucose concentrations rises initially to 7 mM and returns to 5 mM in approximately 40–60 min ( Figure 4C).

***Glucose metabolism***. During the glucose absorptive phase, liver glucose metabolism switches from fasting glucagon-controlled net glucose output, ≈ 0.25 mmoles min ^-1^ (N.B. this has a negative value as glucose exits the liver) to feeding net glucose uptake (a positive value, stimulated by insulin and high GLP-1, where NHGU transiently rises to 1.8 mmoles min ^-1^ ( Figure 3E _i_ and 3E _ii_). These simulations match previously observed hepatic glucose metabolic rates in humans, obtained using the splanchnic/hepatic balance technique ^68^.

The time dependent changes in peripheral insulin-dependent metabolic rates (muscle and adipose tissue are also shown ( Figure 3G). On switching from fasting to feeding with high rates of GLP-1 secretion, there is a large increase in peripheral insulin-dependent metabolism; rising from 0.2 mmoles min ^-1^ during fasting, to a peak rate of 5–6 mmoles min ^-1^ during glucose absorption.

Insulin-independent glucose metabolic rates (brain), change relatively little (from 0.5 to 0.6 mmoles min ^-1^; Figure 3F). As the systemic arterial glucose concentration does not exceed the renal threshold for glucose reabsorption there is no significant glycosuria. These simulations were designed to mirror well-established *in vivo* findings in humans and dogs ^38, 46, 69^.

***Splanchnic and systemic concentration changes in insulin, glucagon and GLP-1.*** Because pancreatic hormones and incretins are directly secreted into the splanchnic circulation and then subject to serial degradation, firstly within the liver sinusoids and then within the peripheral circulation, splanchnic concentrations are normally double those in the systemic circulation, ( Figures 4A–H, Table 1C Insulin equation 4A) ^63^.

### Effects of GLP-1 on blood glucose concentrations and metabolism

Following its glucose-dependent release from enteroendocrine L cells, GLP-1 concentration increases rapidly in splanchnic and systemic circulation ( Figure 4D, H). Release rate depends on the glucose sensitivity coefficient, which is varied from 0.1 to 40 in a geometric progression, ( Table 1D GLP-1 equation 1). GLP-1 sensitizes the hepatic insulin response by AMPK-dependent increases in glucokinase and GLUT2 activity ( Figure 3Ei and 3Eii, Table 1B Glucose equation 5B) and sensitises glucose metabolism to insulin in adipocytes and muscle ( Figure 3G, Table 1B Glucose equation 5A).

The model replicates the observed changes occurring when GLP-1 is released into the splanchnic circulation of normal adults and in GLP-1deficiency when blood GLP-1secretion and concentrations are 0–5% of the controls ( Figure 2– Figure 4) ^70^. With attenuated GLP-1 secretion, the glucose-dependent SMA and PV blood flow rises are decreased ( Figure 2A and 2B); also blood volume redistribution ( Figure 2D and 2E) and portal blood pressure ( Figure 2F). Decreased GLP-1secretion prolongs the splanchnic vascular and metabolic responses to ingested glucose.

Decreased SMA flow in response to glucose absorption, increases splanchnic and systemic glycaemia ( Figure 4G). Peak SM capillary glucose concentration decreases as GLP-1 secretion rate increases, the GLP-1 glucose sensitivity is (K _½GLP-1_=7.2 ± 1.2 range 0–50) giving half maximal reduction in SM capillary glucose from 45 to 22 mM.

In GLP-1 deficient states, although PV glucose concentration is double that with high GLP-1secretion ( Figure 4C and 4G), PV glucose flow (8.4 mmoles min ^-1^) is less than half that found in controls (23 mmoles min ^-1^; Figure 3A). The compensatory rise in HA flow with elevated systemic glucose concentration is double HA glucose flow (14.0 mmoles min ^-1^) found with high rates of GLP-1 secretion (6–8.0 mmoles min ^-1^) and partially compensates for the lower PV glucose flow ( Figure 3C). Thus, peak net hepatic glucose HV outflow (30 mmole min ^-1^) with low rates of GLP-1 secretion is similar to that with high GLP-1 secretion rates (35 mmoles min ^-1^).

In the absorptive phase, hepatic sinusoids avidly accumulate glucose, with high GLP-1 secretion rates the (NHGU is 1.7 mmole min ^-1^; Figures 3E _i_ and 3E _ii_). Because of rapid rates of insulin-dependent hepatic and peripheral metabolism, blood glucose concentrations in both systemic and splanchnic circulations decline rapidly, ( Figure 4C and 4G). With low GLP-1 secretion rates, slower rates of hepatic and peripheral insulin dependent metabolism ( Figures 3E _i_ and 3E _ii_), lead to the high concentrations, more prolonged, systemic and splanchnic glucose concentrations. Thus, in low GLP-1 secreting states, liver is exposed to higher glucose concentrations for a longer and NHGU remains positive for longer than with high rates of GLP-1 secretion.

### Effects of GLP-1 on insulin and glucagon secretion and blood concentrations during fasting and glucose absorption

***Insulin.*** Insulin and glucagon secretion rates are controlled by systemic glucose, unlike GLP-1 and other incretin secretions which are regulated by splanchnic glucose concentrations ( Figure 1, Table 1C Insulin equation 2, Table 1E Glucagon equation 2 *)*. The insulin concentration in splanchnic-capillaries is 2-3-fold higher than systemic blood, ( Figure 4F and 4B). In low GLP-1secretory states, owing to reduced glucose-sensitivity, splanchnic insulin and glucose concentrations are raised. This is mainly due to reduced hepatic, pancreatic and peripheral tissue metabolic sensitivity to insulin, ^71^. In low GLP-1secreting states even with higher blood glucose ( Figure 4C and 4G) and insulin concentrations ( Figure 4B and 4F), both the peak hepatic glucose metabolic rate and peripheral insulin-sensitive glucose metabolism are depressed, ( Figure 3E and 3G).

Systemic insulin concentration (45 pM) during fasting, with low GLP-1secretion rates, is raised by more than 55% above that seen with high GLP-1 (29 pM) secretion rates. In low GLP-1 secreting states in the later prandial period >1–2h after glucose feeding, systemic insulin concentration is > 100% above that seen with high rates of GLP-1, ( Figure 4B and 4F). Splanchnic insulin concentration also is approximately three-fold higher in low GLP-1 than with high rates of GLP-1 secretion at this time. These effects are due to GLP-1-enhanced insulin-dependent metabolic rates that result in decreased blood glucose.

***Glucagon.*** Splanchnic glucagon is approximately double the concentration in systemic blood ^61^. The model simulates this condition in fasting conditions, but shows that during the glucose absorptive phase, when glucagon is at its minimum concentration, the splanchnic/systemic glucagon ratio falls to approximately 1. With low rates of GLP-1secretion, owing to raised systemic glucose concentrations, particularly in the post-prandial phase of digestion > 10min after gavage, systemic and splanchnic glucagon concentrations are decreased ( Figure 4A and 4E).

***Intestinal glucose permeability.*** Intestinal glucose permeability ( Table 1B Glucose equation 1A) is defined as the rate of glucose flow intestinal wall area per unit glucose concentration difference (mM) (mmole s ^-1^ cm ^-2^), between the luminal source and splanchnic capillary sink. Because of the many uncertainties relating to uncontrolled variables, intestinal glucose permeability is not readily determined *in vivo*. The very high rate of glucose uptake from the *in vivo* intestine requires that a known length of intestine be rapidly perfused with high glucose loads to prevent the luminal glucose concentration falling to levels where net flux becomes unmeasurable ^15, 19^. Using a high flow via a triple lumen tube, single pass perfusion over a known length of jejunum, with a “physiological” concentration of isotonic glucose ≅ 350 mM, human glucose “permeability” *in vivo* was estimated at 1×10 ^-3^ cm s ^-1^ ^72, 73^. Absorption was complete in 25–30 min, estimated t _½_= 6.3 min, ^73^.

The Lennernäs protocol does not actually measure the effect of the transmural glucose gradient on net intestinal glucose uptake. This method measures “unidirectional” intestinal permeability, as it ignores any effects of glucose concentration within the mesenteric capillaries, or effects of capillary perfusion on glucose permeability. Because hyperglycaemia induced by intravenous infusion was without measurable effect on human intestinal glucose absorption. It has been assumed there is no significant reflux component to glucose uptake ^74^.

However, during the absorptive phase of digestion, the very high rate of glucose uptake from the intestinal lumen significantly raises the splanchnic vessel glucose concentration to at least twice that of systemic glucose ^75^. Raising the splanchnic capillary glucose reduces the glucose concentration gradient between the intestinal lumen and capillaries. This reduced gradient will reduce intestinal net glucose uptake and hence the unidirectional permeability. It was observed that following intragastric feeding with 1.5g glucose/kg, canine splanchnic glucose balance, i.e. net glucose uptake, was raised to a maximum of 6 mg/min/kg within 30 min and declined after 60 min, reaching a minimum after 120 min. With a higher glucose load (2.5 g/kg), the maximal splanchnic glucose balance still attained 6 mg/min/kg after 30 min, and reached a minimum after 180 min, ^75^. Since the maximal rate of intestinal glucose absorption is the same with both 1.5 and 2.5 g/kg, this indicates that contrary to earlier assumptions, following intraluminal feeding intestinal glucose permeability is slowed by raised splanchnic glucose concentrations.

As previously stated, there are two components to intestinal glucose permeation; Na-dependent glucose cotransport, which because it is very asymmetric ^76, 77^ is insensitive to cytosolic and sub-mucosal glucose concentrations and paracellular glucose permeation, which depends on the glucose concentration gradient existing between the intestinal lumen and the interstitial glucose concentration ( Table 1B Glucose equation 1A). During glucose absorption, intestinal capillary glucose concentration is a function of the following variables: the rates of Na-dependent glucose cotransport and the paracellular glucose permeability coefficient; superior mesenteric arterial flow; the superior mesenteric capillary glucose concentration and the concentration difference between intestinal luminal glucose. Superior mesenteric blood flow is regulated by the GLP-1 concentration, which is in turn regulated by the intestinal luminal glucose concentration. Thus glucose-dependent GLP-1 release generates a feedback control loop which controls SMA flow and the SM capillary glucose concentration.

### Effects of varying the paracellular glucose permeability P _gl_ and GLP-1 secretion on intestinal glucose absorption and metabolism

The effects of variation of the paracellular glucose permeability (0–0.16 μm s ^-1^) and with variable rates of GLP-1 sensitivity glucose sensitivity coefficient (0–100) following a constant initial glucose load = 50 G and constant Na-dependent cotransport rate on the key major model variables are shown in Figure 5– Figure 8 during fasting and a peak rates of glucose absorption. Glucose circulation and its metabolism alter with GLP-1 secretion rates. The controlling coefficient affected GLP-1 secretion is its glucose sensitivity detected by the glucose transporters within enteroendocrine cells.

In Figures 5A–I and Figure 6A–J, the effects of increasing paracellular glucose permeability from 0–0.16 µm s ^-1^ with a range of glucose sensitivities of GLP-1 secretion (2–50) are illustrated using 3D surface contour plots. GLP-1 glucose sensitivity and intestinal glucose permeability P _gl_ are plotted as *x* and *y* coordinates and the dependent variable in the vertical *z* plane. Figure 5A–I shows the dependent variable values during fasting and at peak height during glucose absorption. The peak after feeding occurs within 3–10 minutes after absorption. Increasing P _gl_ from 0 to 0.16 μm s ^-1^ with a constant rate of GLP-1 secretion (= 50) and low pre-sinusoidal (PV) resistance (0.005 mm Hg.s ml ^-1^), results in a hyperbolic increase in portal venous glucose flow from a base of 2.45 mmol min ^-1^ to a maximal flow of 22.3 mmol min ^-1^, the P _gl_ = 0.024 µm s ^-1^ ( Figure 5D).

### Effects of varying the paracellular glucose permeability P _gl_ on blood flows and blood glucose flows

A synergistic response of portal blood flow and glucose flow results from interactions between P _gl_ and GLP-1 secretion. Relatively large changes in superior mesenteric artery, (SMA) flow ( Figure 5A) and portal venous (PV) flow rates ( Figure 5F) occur when both P _gl_ and GLP-1 sensitivity are varied.

Increasing P _gl_ from 0 to 0.16 µm s ^-1^ with low glucose sensitivity to GLP-1 secretion increases the SMA flow from 200 to 315 ml min ^-1^; whereas when GLP-1 sensitivity secretion to glucose is high (= 50), increasing P _gl_ from 0 to 0.16 µm s ^-1^ increases SMA flow from 450 to 1150. The P _gl_ giving half maximal activation of SMA flow remains unchanged at 0.02 µm s ^-1^.

PV glucose flows also increase hyperbolically on increasing P _gl_ ( Figure 5C). Glucose flow is substantially higher ( *21 mmole min ^-1^*) when both P _gl_ and GLP-1 are high, than with high GLP-1 and P _gl_ = zero ( *PV glucose flow increases from 1.9–2.5 mmole min ^-1^*. *When GLP-1 secretion rates are low GLP-1 _gl_. _sens_ = 2, increasing P _gl_ from 0 to 0.16 µm s ^-1^ PV glucose flow increases only 11.7 mmole min ^-1^*).

Increasing P _gl_ from 0–0.016 µm s ^-1^ increases glucose flow rates from the intestinal lumen resulting in a hyperbolic rise in splanchnic and systemic circulation glucose concentrations ( Figure 5H and 5D).

As already shown in Figure 4C and 4G, when GLP-1 glucose sensitivity is increased (2–50) maximal splanchnic glucose concentration decreases linearly from 37 to 19 mM; systemic glucose remains at approximately 15 mM ( Figure 5D).

The relative insensitivity of systemic compared with splanchnic glucose concentration to changes in GLP-1 secretion, can be ascribed to the relative constancy of HV glucose outflow into the systemic circulation.

### Synergism between paracellular glucose permeability and GLP sensitive SMA flow

With low rates of GLP-1 secretion and high P _gl_ SM capillary glucose concentration is raised during the absorptive phase to 37 mM ( Figure 5H). With increasing rates of GLP-1 secretion SM capillary glucose falls to 9.0 mM; *(K _½_ = 0.018 µm s ^-1^ falling to 0.013 µm s ^-1^ when GLP-1 secretin = 50).* During fasting periods ( Figure 5I) altering P _gl_ is without any effect on either splanchnic or systemic glucose concentration.

The observed unidirectional glucose permeability rises during the absorptive phase of glucose digestion and reaches a maximum about 2–4.0 min after initial exposure to luminal glucose feeding ( Figure 3D). In Figure 6A with low rates of GLP-1 secretion, the peak intestinal glucose permeability increases as a hyperbolic function of P _gl_ *(K _½_ = 0.02 µm s ^-1^).* On increasing GLP-1from 2 to 50, the maximal observed permeability P _gl_ increases from 0.041 to 0.056 µm s ^-1^; (K _½_= 0.03 µm s ^-1^). Thus owing decreased SM capillary glucose resulting from higher rates of SM capillary perfusion the concentration gradient between the intestinal lumen and the submucosal capillaries thereby increasing paracellular glucose diffusion. Consequently there is a positive interaction between GLP-1 secretion and intestinal paracellular permeability ( Figure 6A).

These simulations explain why apparently contradictory results on intestinal glucose permeability have been reported. In T2DM subjects compared with controls, no change in intestinal glucose uptake is observed when intravenous glucose and insulin are clamped, ^78^. Whereas in critically ill patients with a lower SMA response to glucose infusion, irrespective of their GLP-1 secretory status, the intestinal absorption rate is decreased ^79^.

### Effects of altered paracellular permeability and GLP-1 on hepatic and peripheral glucose metabolism

When splanchnic blood glucose is abundant during the absorption, increasing both GLP-1 secretion and intestinal glucose permeability P _gl_, synergistically increase NHGU ( *c = 6.08*), and insulin dependent peripheral glucose metabolism ( *c* = 14.6) ( Figures 6B–D). NGHU increases as a hyperbolic function of increasing P _gl_, ( Figure 6B). Systemic insulin ( Figure 6E) and GLP-1 concentration ( Figure 6H) also increase with increasing P _gl_, (K _½_ ≈ 0.03 µm s ^-1^). The reciprocal changes in insulin-dependent and insulin-independent metabolism ( *c* = -13.55) ( Figure 6D), result from the more intense competition for systemic glucose from insulin dependent tissues.

During fasting, increasing P _gl_ and/or rates of GLP-1 secretion do not synergise blood flows or glucose flows ( Figures 5B, 5G and Figure 6H). When the intestinal lumen is empty, increasing P _gl_ has no effect on systemic or splanchnic glucose ( Figure 5E and 5I), whilst increasing glucose sensitivity of GLP-1 secretion only results in small increases in GLP-1 release or SMA flow; thus interaction between P _gl_ and GLP-1 in zero i.e. (c ≅ 0).

### Effects of altered paracellular permeability on insulin, glucagon and GLP-1 secretion and blood glucose concentrations

During the intestinal glucose absorptive phase, positive interactions occur between P _gl_ and glucose sensitive GLP-1 secretion on GLP-1 and insulin concentrations within splanchnic and peripheral blood Figure 6E, J, H and I). Because glucagon secretion decreases as systemic glucose increases, negative interactions occur between GLP-1 and intestinal P _gl_ on glucagon secretion and concentrations. The interaction coefficients are for splanchnic ( Figure 6F; *c = 0.06)* and systemic glucagon ( *c = -0.99*; ( Figure 6F, G). In the absence of intestinal glucose absorption zero interaction takes place between GLP-1 secretion and P _gl_ on splanchnic glucose concentrations.

## Part 2 Simulations of NAFLD, NASH and T2DM

### Portosystemic shunting

Normally, direct blood flow between the portal vein and hepatic vein is prevented by a high intrahepatic portosystemic resistance. Trans-hepatic blood flow resistance is normally very low and portosystemic shunt (PSS) resistance is very high, so 99.0% of portal venous glucose during peak absorption flows via the sinusoids. However, in conditions such as hepatic cirrhosis and/or hepatosteatosis, increased tortuosity of hepatic sinuses and narrowing of the hepatic vessels results in development of low resistance intrahepatic collateral vessels enabling portosystemic shunt PSS flows ^80^. Two important effects of hepatic and portal endothelial dysfunction are increased hepatic vascular resistance resulting in reduced hepatic sinus blood flow and raised portal blood pressure ^81– 83^. Additionally, reduction in hepatic glucokinase activity, associated with NASH and T2DM, reduces hepatic insulin-and GLP-1-dependent glucose uptake and metabolism ^84, 85^.

Glucose passing through the sinusoids is processed initially by hepatocyte GLUT2 and glucokinase activities. Both these activities are regulated by insulin and GLP-1 ^86, 87^. Although intrahepatic PSS formation alleviates portal hypertension ^88, 89^, it also circumvents metabolic processing in liver sinusoids, with adverse consequences on glucose, insulin, glucagon and incretin circulation and metabolism. Splanchnic blood contents enter the systemic circulation directly via the PSS, particularly during the absorptive phase of digestion and thereby raise systemic concentrations of glucose, insulin, glucagon and GLP-1inappropriately, (see below).

Prolonged hyperglycaemic exposure of splanchnic endothelia could result in mitochondrial starvation of ascorbate ^90, 91^ which could be either an initiating or exacerbating cause of NASH.

The model of glucose absorption is used here to test a range of portosystemic shunt resistances from 40 to 0.005 mm Hg.s ml ^-1^. With a presinus resistance = 0.005 mm Hg.s ml ^-1^ the change in shunt flow varies as a hyperbolic function, from zero with high shunt resistance to 560 ml min ^-1^ with low resistance, *(V _max_ = 1160 ml min ^-1^; K _½_= 0.11 mm Hg.s ml ^-1^* with high presinusoidal resistance = 0.025 mm Hg.s ml ^-1^, the estimate of maximal shunt flow increases to 2034 ** ml min ^-1^ *; (K _½_= 0.43 mm Hg.s ml ^-1^).* Portal hypertension as seen in hepatic cirrhosis and NAFLD/NASH is associated with increased hepatic vascular resistance. The model simulates “portal vein resistance” by raising pre-sinusoidal hepatic resistance from 0.005 to 0.025 mm Hg.s ml ^-1^. The higher pre-sinusoidal PV resistances give comparable changes in portal vein pressure to those observed in animal models of NAFLD ^92^.

Although others have modelled metabolic syndrome in relation to glucose metabolism to date no other simulation model incorporates portosystemic shunt flows into models of NASH and T2DM, ^93, 94^.

### Low incretin secretion during NAFLD, NASH and T2DM

It has been suggested that incretin secretion and/or responses to incretins are defective in obesity, NAFLD, or T2DM, ^70, 95– 97^. The improvements in patient glycaemic responses elicited by GLP-1 agonists, or dipeptidyl peptidase inhibitors that retard GLP-1 degradation, within the circulation, or AMPK activators, e.g. metformin or other biguanides ^40, 64, 98^ and reversal of the pathological effects of NAFLD and NASH by GLP-1 agonists ^85, 99^ tend to corroborate the view that GLP-1 deficiency is a cause of metabolic disease. GLP-1 agonists, such as exenatide, used in treatment of 2TDM, have been shown to be effective in reducing hyperglycaemia and hyperinsulinaemia namely ^100^. Several reports indicate that incretin deficiency in T2DM and in morbid obesity may be partially reversed by bariatric surgery with subsequent weight loss ^87, 96– 98^.

Nevertheless, other reports show an absence of correlation between GLP-1 secretion and obesity ^64^, and it is evident that T2DM may occur without any marked deficit in GLP-1 secretion ^99^. Thus it seems that low GLP-1 secretion rates observed in NASH, or in T2DM may be a consequence of the changes in glucose metabolism, rather than a cause. Thus modelling the mechanical effects of portosystemic shunting and increased presinusoidal resistance on blood, glucose, hormone and incretin circulation and metabolism may be useful in elucidating the role of GLP-1secretion in metabolic disease syndrome, with or without PSS.

### Simulation of the effects of raised pre-sinus resistance and portosystemic shunting on blood volumes, flows and pressures

The effects of varying PSS resistance from high resistance (10 mm Hg.s ml ^-1^), where virtually zero shunt blood flow occurs, to low resistance (0.005 Hg.s ml ^-1^), where approximately 50% of portal blood flow is shunted, on the time courses of change in insulin, glucagon and GLP-1 concentrations in splanchnic and systemic blood following glucose gavage is described in ( Figure 7 and Figure 8). The fraction of splanchnic blood flow diverted via the PSS is similar to that when PSS has been surgically initiated by transjugular intrahepatic portosystemic shunting, TIPS ^80, 82, 101^. The hepatic presinus resistance i.e. trans-hepatic blood flow resistance is maintained at a high level 0.020 Hg.s ml ^-1^ (4× higher than the control value = 0.005 Hg.s ml ^-1^ used in Part 1). These simulations are consistent with those found in NASH ^83, 102^.

### Blood flow effects

PSS blood flow decreases as a hyperbolic function of increasing shunt resistance. *The PSS resistance giving half maximal flows, (K _½_ = 0.025 Hg.s ml ^-1^) where V _max_ of shunt flow, is 600 ml ^-1^* ( Figure 7A). PSS blood flow peaks when SMA flow and PV pressure are maximal ( Figure 7E and 7C) and returns to fasting rates once intestinal glucose absorption is completed. PSS flow falls rapidly from its peak to fasting level *(t _½_ ≈ 5 min)*. Peak PV flow falls reciprocally as PSS rises ( *K _½_ = 0.028 Hg.s ml ^-1^; maximal PV flow 725 ml ^-1^).* PV flow decreases from its peak at a slightly slower *rate, (t _½_ ≈ 7.5 min to reach a plateau phase*) ( Figure 7C). During this plateau phase PV flow also decreases as a hyperbolic function of PSS resistance *(K _½_ = 0.028 Hg.s ml ^-1^;* Figure 7C).

### Effects of presinusoidal resistance and portosystemic shunting on splanchnic blood flows and pressure

The primary effect of reducing the PSS resistance clearly is to increase PSS flow. However this flow is also modulated by the presinusoidal resistance. When shunt flow is negligible *(PSS resistance ≥ 0.4 Hg.s ml ^-1^)* increasing presinusoidal resistance from the normal low resistance = 0.005 Hg.s ml ^-1^ to the high resistance, as found in NASH, portosystemic shunt blood flow increases by only a small amount, from 13 ml min ^-1^ to 90 ml min ^-1^. But with low PSS resistance = 0.005 Hg.s ml ^-1^(shunt open); raising presinusoidal resistance from 0.005–0.025 Hg.s ml ^-1^ raises shunt flow from 557–1600 ml min ^-1^. There is evidently a strong interaction between PSS and presinusoidal resistance on hepatic shunt flow. When GLP-1 secretion rates are high, reducing the PSS resistance below 0.027 Hg.s ml ^-1^ reduces peak PV pressure by 50% ( Figure 7I). Thus with high presinusoidal resistance and high rates of GLP-1 secretion portosystemic shunting diverts ≈ 80% of the portal blood flow away from the sinusoids. Reduction in either PV resistance, or PSS resistance reduces peak shunt flow and reduces portal venous pressure ( Figure 7G).

### Effects of portosystemic shunting on glucose flow and blood concentrations

Following duodenal glucose gavage, glucose flow via the PSS rapidly reaches a peak (2–3min), *(maximal flow 14.5 mmole min ^-1^; t _½_ ≈ 1.5 min, K _½_ = 0.028 Hg ml s ^-1^; Figure 7B)*. PV glucose flow has peak of approximately 20 mmole min ^-1^ and decreases hyperbolically with PSS resistance ( Figure 7D). ** Hepatic arterial blood flow and HA glucose flow decrease during the initial stages of glucose absorption from 720–650 ml min ^-1^ ( Figure 7F). Hepatic shunt flow has no significant effect on HA flow.

### Effects of portosystemic shunting with high pre-sinusoidal resistance on glucose metabolism

GLP-1 secretion causes a large increase in insulin-dependent metabolism in liver and muscle and adipose tissues ( Figure 3D–F). Opening the PSS resistance <0.05 Hg.s ml ^-1^ reduces the effect of GLP-1 on hepatic glucose metabolism ( Figure 8D). With high PSS flows, net hepatic glucose uptake, NHGU, switches more quickly to glucagon-activated gluconeogenesis as the negative values in NHGU *(8–14 minutes after the start of glucose gavage,* synchronously with the second peak in shunt glucagon flow ( Figure 8C).

In control subjects after duodenal glucose gavage, insulin release stimulates hepatic glucose consumption, ( *peaking 4–6 min after gavage*) ( Figure 3E). With a large PSS, even with high rates of GLP-1 secretion, both hepatic and peripheral insulin-dependent glucose consumption peaks are much reduced ( *PSS K _½_ = 0.02 Hg.s.ml ^-1^*), and occur sooner after glucose gavage, ( *3–5 minutes*) ( Figure 8D and 8E). With high PSS flows, when systemic and splanchnic glucose concentrations fall to lower levels ≈ 2 mM and insulin-independent glucose metabolic rates are reduced, ( Figure 8F, Figure 10B and Figure 10F).

### Effects of portosystemic shunting with raised presinusoidal resistance on insulin, glucagon and GLP-1 flows

GLP-1, insulin and glucagon flows after duodenal glucose gavage with varying PSS resistance are shown in Figures 8A–C. GLP-1 flow via the PSS rises swiftly when glucose is absorbed from the intestine into the splanchnic circulation; ( *PSS R giving half maximal GLP-1 flow is 0.027 Hg.s ml ^-1^*) ( Figure 8A). *Peak flow occurs approximately 3 mins after the start of duodenal glucose gavage and decreases very rapidly thereafter (t _½_ ≈ 3 min).* PSS resistance change has only a small effect on GLP-1 flows and on systemic blood concentrations after the initial surge in GLP-1flow; *(with zero PSS shunting, systemic blood GLP-1= 1.9 nM, and with maximal shunting, splanchnic GLP-1= 1 pM*; Figure 10A and 10E); ( *with open shunting during fasting splanchnic GLP-1= 3.5 pM and with zero shunting, GLP-1= 6.6 pM).* During fasting and in the late post-absorptive phase, with an open PSS, systemic GLP-1 concentration is approximately 30% of that with zero PSS shunting ( Figure 10E). This reduced GLP-1 resulting from PSS, could explain the low plasma GLP-1 levels reported in metabolic disease syndrome ^64, 96, 97^.

***Insulin***. Insulin flow via the PSS peaks 2.5–3min after the start of glucose gavage ( Figure 8B) *. The shunt resistance giving half maximal peak insulin flow (K _½_ = 0.063 Hg. s ml ^-1^) is twice as high as that required to give half maximal shunt flows of GLP-1, or glucagon. Insulin flow via the shunt decreases rapidly from its peak value (t _½_ ≈ 3 min, but is sustained for longer t _½_ ≈ 15 min as the shunt resistance is reduced. * A second wave of insulin peaks 16–20 min after the start of glucose gavage.

Shunting has complex effects on both systemic and splanchnic blood insulin concentrations. The most striking effect being the sustained increase in systemic plasma insulin during fasting and in the absorptive phase ( Figure 10Gi and 10Gii) and the large decrease splanchnic insulin concentration observed shortly (2–7 min) after glucose gavage ( Figure 10C).

***Glucagon***. Following glucose gavage, two waves of glucagon flow via the PSS are evident when shunt resistance is ≤ 0.015 Hg. s ml ^-1^. The first wave peaks at 1–2 min *, at a flow rate of 20 fmoles min ^-1^ and rapidly decreases; (t _½_ = 1.5 min)* ( Figure 8C). The second larger glucagon flow wave peaks at 38 fmoles min ^-1^, 8–10 min after gavage. *This flow is half maximal when shunt resistance is ≈ 0.055 Hg. s ml ^-1^ but is sustained at (10–20 fmoles min ^-1^) at least 20 min after gavage (t _½_ = 10–15min)*.

Hyperglucagonaemia is often linked with 2TDM ^70, 96, 103, 104^ and importantly has been observed with normal GLP-1 secretion rates when portosystemic shunting is present, due to hepatic cirrhosis, ^104^.

### Effects of raised pre-sinus resistance and portosystemic shunting on unidirectional intestinal glucose permeability

A consequence of PSS-dependent stimulation of insulin-dependent glucose metabolism is reduced systemic and splanchnic capillary glucose concentration ( Figure 10A and 10B). This steepens the glucose concentration between intestinal lumen and SM capillaries and thereby increases the unidirectional glucose permeability ( Figure 8G). A similar increased rate of intestinal glucose uptake in diabetic patients is observed following metformin treatment ^105^.

These increases in unidirectional rates do not signify real change of intestinal permeability. Nevertheless, real increases in intestinal permeability may occur as a result of splanchnic oedema following portal hypertension ^106– 108^.

### The effects of portosystemic shunting on the rates of insulin, GLP-1 and glucagon secretion

The time course of insulin, glucagon and GLP-1 secretion rates are demonstrated as functions the GLP-1 glucose sensitivity as controls, without shunting and normal low presinusoidal resistance ( Figure 9A, 9C and 9E), and with portosystemic shunting and high presinusoidal resistance, as obtains in NASH ( Figure 9B, 9D and 9F). Insulin secretion rates increase during the glucose absorptive phase of metabolism. This increase is stimulated directly by systemic glucose concentration affecting pancreatic beta cells insulin production ( Figure 1, Insulin equation 1) and by the glucose sensitivity of GLP-1 secretion ( Table 1E GLP-1 equation 1; Figure 9A).

During fasting, insulin secretion rates are directly proportional to GLP-1 glucose sensitivity, however during peak glucose absorption, insulin secretion rates are less GLP sensitive. With low rates of GLP-1 secretion, systemic glucose is raised and compensates in part for reduced GLP-1 glucose sensitivity of insulin release.

GLP-1 secretion has a similar time course to that of insulin, Figure 9C. GLP-1 secretion has a hyperbolic dependence on glucose sensitivity of GLP-1 secretion cells during fasting. During fasting glucose generated by glucagon-stimulated gluconeogenesis ( Figure 8D and Figure 9E) raises GLP-1 secretion. Shunting causes a rapid decay in the initial peak of GLP-1 secretion due to the sharp decrease in splanchnic glucose that occurs almost immediately following glucose gavage. Low GLP-1 secretion rates diminish the effects of shunting on metabolism and excessive glucagon release.

### Portosystemic shunting alters the timing and extent of insulin, GLP-1 and glucagon release relative to changes in systemic and splanchnic glucose

It is evident that glucose, insulin, GLP-1 and glucagon leakages via the PSS alter the normal balance between glucose supply and its disposal in the splanchnic and systemic circulations. The changes in systemic and splanchnic glucose, insulin, glucagon and GLP-1 are shown in Figure 10. The most obvious effects of shunting are displayed in Figure 10C, 10D and Figure 10F, 10G and Figure 10H.

Peak systemic glucose ( Figure 10F; *PSS resistance K _½_= 0.05 Hg.s.ml ^-1^)* and splanchnic insulin *(PSS resistance K _½_= 0.145 Hg.s.ml ^-1^*; Figure 10C) are decreased by shunting 5 min after duodenal gavage. The decrease in splanchnic insulin coincides with a shunt-dependent increase in systemic and splanchnic glucagon ( Figures 10D, 10H). Portosystemic shunts increase fasting systemic insulin concentrations ( Figure 10G) *(PSS resistance K _½_= 0.06 Hg.s.ml ^-1^)*.

Systemic and splanchnic glucagon concentrations have very large responses to opening the portosystemic shunt ( Figure 10D and 10H). In addition to a peak 10 min after gavage *(PSS resistance K _½_= 0.06 Hg.s.ml ^-1^)* a second sustained rise in both systemic and splanchnic glucagon is evident *(PSS resistance K _½_= 0.075 Hg.s.ml ^-1^)*.

### Effects of shunting on normalized systemic and splanchnic insulin; GLP-1; or glucagon/glucose ratios.

The extent to which the shunt leakages affect metabolism is reflected in altered rates of insulin-dependent peripheral glucose metabolism. This is evident from the change in peripheral insulin dependent metabolic rate relative to systemic glucose concentration. Figure 11D and of hepatic glucose metabolic rate relative to splanchnic glucose concentration Figure 12D.

Normalizing the systemic and splanchnic hormone and incretin concentrations relative to glucose concentrations in the appropriate compartments illustrate more precisely the specific effects of shunting.

The simulated data obtained with PSS are normalized relative to the ratios in the absence of PSS (i.e. with a portosystemic resistance = 40 mm Hg.s ml ^-1^). The normalized ratios obtained show the excess or deficit in hormone or incretin response relative to glucose as function of portosystemic shunt opening. In the fasting state opening the shunt *(from 40 to 0.005 mm Hg.s ml ^-1^*) increases the normalized insulin: glucose ratio to 2.1 above control (without shunting) *(K _½_ = 0.03 mm Hg.s ml ^-1^)* ( Figure 11A and Figure 12A).

The normalized systemic insulin: glucose ratio increases as shunt resistance falls to a maximum of 5.4 ( *PSS R K _½_ = 0.03–0.04 mm Hg.s ml ^-1^).* Two peaks in the systemic insulin: glucose ratio ( Figure 11A) and in splanchnic insulin/glucose ratio ( Figure 12A). The second smaller, but longer lasting increase in the insulin/glucose ratio, coincides with the second wave in hepatic gluconeogenesis/glucose ratio ( Figure 12E; *PSS R K _½_ = 0.06 mm Hg.s ml ^-1^*) and peripheral insulin-dependent metabolism ( *PSS R K _½_ = 0.015 mm Hg.s ml ^-1^*; Figure 11D). The ratio of systemic GLP-1/glucose also increases during the early phase of glucose absorption when the PSS is opened ( *PSS R K _½_ = 0.028 mm Hg.s ml ^-1^*; Figures 11B and Figure 12E).

### Effects of shunting on normalized systemic and splanchnic insulin/GLP-1-dependent metabolism/glucose ratios

GLP-1 and insulin synergistically stimulate systemic glucose metabolism in insulin-sensitive tissues. Plots of the product of the normalized (GLP-1 **x**insulin product)/Glucose ratios ( Figure 11E and Figure 12D) show large and inappropriate stimulation of peripheral insulin-dependent glucose metabolism ( Figure 11E) and also stimulus to hepatic metabolism when the PSS is open ( Figure 12D). Similar findings have been observed in the adipose tissues of patients with NASH ^109^. PSS–dependent stimulus to insulin metabolism also causes a very large increase in hepatic metabolism relative to splanchnic glucose, particularly during the glucose absorptive phase. However this stimulus continues at a lesser level during the later digestive periods ( Figure 12E).

## Discussion

 The chronology of events resulting from PSS following glucose gavage assists understanding of the complex interactions induced by hepatic shunting and are outlined in Figure 13:

• Figure 13A. Shunt flows of GLP-1 and glucose and to a lesser extent insulin, are the earliest expression of PSS-dependent alterations in flow and metabolism (0–10min).
• Figure 13A and 13B. A very large (tenfold) increase in the insulin-sensitive glucose metabolism in muscle and adipose tissue follow. This is accompanied by fall in systemic glucose concentration ( *red continuous line)* relative to that observed in controls without shunting.
• Figure 13C. The shunt condition decreases systemic and splanchnic glucose, raises glucagon secretion and inhibits GLP-1 secretion. Insulin secretion is also raised.
• Figure 13D and 13E. The early onset of hypoglycaemia with high PSS increases splanchnic glucagon, thereby increasing hepatic gluconeogenesis. This promotes partial recovery of systemic glucose and insulin concentrations.
• The oscillations of peripheral insulin dependent metabolism and splanchnic gluconeogenesis induced by PSS insulin flow are the cause of hyperglucagonaemia, frequently observed in NASH and T2DM ^70, 96, 104^.

Unanticipated findings of the model simulation of PSS are explanations for the suppression of post-prandial GLP-1 and raised blood glucagon concentrations ( Figure 13C). Reduced GLP-1 concentration in the systemic circulation has been frequently reported, but generally ascribed to intrinsic failure of the secretory process ^96^, rather than as a consequence of splanchnic hypoglycaemia brought about by overstimulation of peripheral insulin-dependent metabolism as demonstrated here.

The model simulation showing that portosystemic shunting in NASH and NAFLD generates imbalances between splanchnic and systemic distributions of insulin, glucagon and GLP-1 relative to glucose that stimulate insulin-sensitive metabolism in both liver that leads to hyperglucagonaemia and low GLP-1 is novel. A recently published clinical paper ^70^ contains results that enable testing of these model predictions.

The hormone/glucose ratios in NAFLD patients ( Figure 14A) and T2DM patients ( Figure 14B) have been obtained from published data of plasma insulin, glucagon, GLP-1 and glucose concentrations ^70^. The time series of insulin/glucose, glucagon/glucose and GLP-1/glucose concentration ratios after OGTT are normalized to those of control subjects. Insulin/glucose ratios exceed those in controls, initially by eightfold and remain higher throughout the test. This finding closely resembles the simulations with moderate portosystemic shunt and raised portal vein resistance shown in Figure 11A and 11C. Glucagon/glucose ratios exceed controls (by 2–3 fold) during the first 100 min of the test meal in both NAFLD and T2DM. As the authors suggest the absence of raised insulin/ratio indicates that insulin secretion may be suppressed in T2DM although not in NAFLD ^70^.

## Summary of model findings

The computer model of human glucose absorption and metabolism demonstrates that increased superior mesenteric arterial (SMA) blood flow following intestinal glucose gavage, synchronous with glucose absorption, and insulin and GLP-1 secretion into the splanchnic circulation, is crucial to a harmonious balance between intestinal glucose absorption and its distribution and metabolism. Raised GLP-1 dependent splanchnic capillary flow, raises the passive component glucose absorption. GLP-1 and insulin synergise net hepatic glucose uptake (NHGU). When GLP-1 secretion is low, retarded SMA flow raises portal venous glucose concentration. Splanchnic hyperglycaemia slows passive glucose diffusion from intestine to capillaries.

A second key factor causing hyperglycaemia is reduced NHGU due to decreased GLP-1-dependent hepatic glucokinase activity. Hyperglycaemia is sustained by reduced synergy of GLP-1 with insulin-sensitive muscle and adipocyte glucose metabolism.

NASH initiates intrahepatic portosystemic shunting. Since splanchnic glucose, insulin and glucagon bypass hepatic sinusoids, this leads to inappropriate stimulation of peripheral insulin-dependent metabolism. This in turn accelerates the decrease in both systemic and splanchnic glycaemia. Splanchnic and systemic hyperglucagonaemia and suppression of GLP-1 secretion follow. Prolonged hyperglucagonaemia results in excess gluconeogenesis resulting in fasting hyperglycaemia and hyperinsulinaemia.

Low rates of GLP-1 secretion could have a protective role in reducing post-prandial portal hypertension. This will also reduce portosystemic shunting of insulin and glucose lower splanchnic hypoglycaemia. Splanchnic hypoglycaemia by stimulating ghrelin release may be a contributory factor in the hyperphagia commonly associated with 2TDM inducing behaviour ^110, 111^.

Prolonged exposure of splanchnic endothelia to hyperglycaemia as occurs with low rates of GLP-1 secretion, could result in mitochondrial starvation of ascorbate due to competition inhibition of dehydroascorbate transport ^90, 91^ and initiate or exacerbate NASH.

## Data availability

The data referenced by this article are under copyright with the following copyright statement: Copyright: © 2016 Naftalin RJ

Data associated with the article are available under the terms of the Creative Commons Zero "No rights reserved" data waiver (CC0 1.0 Public domain dedication). 

*F1000Research*: Dataset 1. Raw data for ‘A computer model simulating human glucose absorption and metabolism in health and metabolic disease states’, 10.5256/f1000research.8299.d117393 ^112^

## Software availability

Jmadonna sims of glucose metabolism-RJN March 2016.

A working copy of the program, with a choice of graphical outputs available for all of the variables displayed in this paper and many others in addition. A trial copy of Berkeley Madonna is available which will permit the program to be run http://www.berkeleymadonna.com/jmadonna/jmadrelease.html without saving the data.
