# Supplementary material for: A computer model simulating human glucose absorption and metabolism in health and metabolic disease states
Source: F1000Res. 2016 Apr 12;5:647. [Version 1] doi: 10.12688/f1000research.8299.1 (PMC4909112; doi:10.12688/f1000research.8299.1)
Supplement: Peer review history

# Peer review: Referee response for version 1

**DOI:** 10.5256/f1000research.8925.r14320

**Published:** 2016-06-10

**Authors:** C Charles Michel

**Affiliations:** 1 Department of Bioengineering, Imperial College London, London, UK

For much of the second half of the twentieth century, the absorption of nutrients from the small intestine was regarded as largely a question of understanding the detailed mechanisms of epithelial transport. That other factors might be involved was suggested by the high rates of glucose absorption from the small intestine of unanaesthetised mammals (including humans) that greatly exceeded those estimated or predicted from in vitro studies An analysis of the transport processes between the epithelial brush border and the blood flowing through the villus capillaries drew attention to the importance of the increased blood flow which accompanied glucose absorption ^1^. This maintained gradients of glucose concentration through the mucosa sufficient to sustain the high rates of glucose uptake. At the time, the connection between intestinal glucose absorption and villus blood flow was unknown but the discovery and isolation of the glucagon like peptide, GLP-1, has led Professor Naftalin to suggest that this signalling molecule is the missing link, in addition to its other roles in glucose metabolism. From this starting point in the present paper, he develops a model

 of glucose absorption which is co-ordinated with changes in blood flow to both the jejunum and the liver and also the insulin dependent uptake of glucose in the liver, adipose tissue and skeletal muscle.

 From this simulation of the regulation of glucose absorption and metabolism in the healthy human, he uses his model to explore changes in occurring in patients suffering from non-alcoholic steatohepatitis, non-alcoholic liver disease and type 2 diabetes mellitus.

 While there are well known models of glucose metabolism ^2^, to my knowledge, this is the first to incorporate intestinal glucose absorption. The way in which the different steps are coordinated here indicates a substantial advance in our understanding for which Professor Naftalin is to be applauded. I believe others will follow his lead and develop his model. It is interesting to see that it accounts successfully for the interactions between the portal and systemic circulations of the liver by simply assigning them with different compliances and different flows. I was a little surprised, however, that the model ascribed so much of the vascular regulation to GLP-1 when GLP-2 is both released concomitantly with GLP-1 and, in some mammalian species including humans, is known to act as a vasodilator in duodenum and ileum, the sites of glucose absorption. There is also evidence that GLP-1 and GLP-2 act synergistically in promoting glucose absorption ^3^. Also, the governing equations of the present model involve many simplifications. For example, the equation for glucose absorption assumes the passive component is entirely accounted for by diffusion and lies in parallel with the active component when there is evidence for passive transport by solvent drag ^4^ and interaction between active and passive components ^1^. These, however, are relatively minor criticisms when the real achievement of the model is its demonstration of the importance of integrative physiology.

 I should say that I did not find this an easy paper to read. This is probably because I believe that the most important part of a paper describing a mathematical (or computer) model of a physiological process lies in the model's governing equations. When the governing equations are set out and justified in a “methods” or “theory” section, the reader can appreciate the simplifying assumptions and the extent to which they might compromise the model's predictions more quickly. Also, I believe, the values used for the model's parameters should be justified by references to the literature, wherever possible. In the present paper, the governing equations are listed in a table with little justification for their general form. The values taken for the constants and parameters are also presented in a table. I appreciate that the modern trend of many journals to relegate mathematical argument to appendices, may increase the readership of a paper and its number of citations but by doing so, it makes the true evaluation of the work more difficult. For this particular paper, however, I believe the additional effort is well worthwhile.

I have read this submission. I believe that I have an appropriate level of expertise to confirm that it is of an acceptable scientific standard.

## References

[1] PappenheimerJRMichelCC: Role of villus microcirculation in intestinal absorption of glucose: coupling of epithelial with endothelial transport. *J Physiol*.2003;553(Pt 2) : 10.1113/jphysiol.2003.043257 561-74 10.1113/jphysiol.2003.043257 12937296PMC2343570

[2] BergmanRN: Orchestration of glucose homeostasis: from a small acorn to the California oak. *Diabetes*.2007;56(6) : 10.2337/db07-9903 1489-501 10.2337/db07-9903 17526912

[3] DruckerDJYustaB: Physiology and pharmacology of the enteroendocrine hormone glucagon-like peptide-2. *Annu Rev Physiol*.2014;76: 10.1146/annurev-physiol-021113-170317 561-83 10.1146/annurev-physiol-021113-170317 24161075

[4] PappenheimerJRReissKZ: Contribution of solvent drag through intercellular junctions to absorption of nutrients by the small intestine of the rat. *J Membr Biol*.1987;100(2) :123-36 343056910.1007/BF02209145

# Peer review: Referee response for version 1

**DOI:** 10.5256/f1000research.8925.r14254

**Published:** 2016-06-09

**Authors:** Ian David Lockhart Bogle

**Affiliations:** 1 Department of Chemical Engineering, University College London, London, UK

This is a comprehensive piece of modelling work.  A complex model has been developed and tested and seems to support the evidence, particularly the effects of porto-systemic shunting which I don’t believe has been included in other modelling studies.  In this sort of work it is difficult to provide unequivocal confirmation of model validity because of the complexity, the fact that some phenomena are inevitably not included although key ones have been, and any clinical data tends to be partial and there may be other things happening that have not been recorded.  However as far as I can see the model supports expected behaviour and reported studies.

 The equations are logical and solution methods seem appropriate.  I have a few comments about parameter sensitivity and equation and parameter presentation below.

 The Conclusions are supported by the predictions.

 I did find it difficult to relate the discussion with the model because of the way that nomenclature was used.  For example on page 15 para 2 it refers to Table 1D GLP-1 equation 1 but I am not clear exactly which parameter it is referring to (similarly four lines later).  What would really help would be for Table 2 to also give the variable name and equation number where it appears for each parameter.

 This table does not give sources for the data and whether they are either taken from literature or fitted. I think this would also help.

 A few sensitivity studies have been done. I think they have been done because they are thought by the author to be important for clinical reasons.  But it may be that model outputs are very sensitive to some parameters and it would be useful to know which ones these are. For example I would have thought that ‘GLUT2 Km’ (presumably ‘KM_GLUT2’ in the model = 20mM) was important given the key role of GLUT2 but sensitivity to this one isn’t tested.  Are there others that are critical to model performance? If so the quality of the data supporting this would be critical. On page 17 ‘portosystemic shunt resistances’ were varied but what is the variable name and equation number where it appears?

 Some of the verification is vague.  For example on p20 it says ‘these simulations are consistent with those found in NASH’ but does this mean qualitatively, in which what particular features, or quantitatively, in which case this could be quantified?  Similarly in the following column ‘Hepatic shunt flow gas no significant effect on HA model’ but this isn’t shown.  Is this to be expected?

 On page 28 does the tenfold increase in insulin-sensitive metabolism apply to the whole metabolism?  If so what is the effect on measurable metabolites and is there evidence that this might be the true?

 Some of the syntax of the models isn’t correct: Renal urine glucose flow (eqn 6 and repeated at the bottom of the page) is missing a bracket and Hepatic glucose metabolic rate has a spare one.  These were the only two I spotted but it is worth checking all.

I have read this submission. I believe that I have an appropriate level of expertise to confirm that it is of an acceptable scientific standard.

# Peer review: Referee response for version 1

**DOI:** 10.5256/f1000research.8925.r13996

**Published:** 2016-05-26

**Authors:** Martin Diener

**Affiliations:** 1 Institute for Veterinary Physiology and Biochemistry, University of Giessen, Giessen, Germany

This is a nice piece of work in which the author simulates elegantly the effect of an incretin, GLP-1, on glucose absorption, metabolism and blood circulation as well as the level of hormones relevant for the regulation of sugar metabolism such as insulin and glucagon. Overall the paper is well written; title, abstract and conclusions are sound.

 I have only some minor comments for improvement of the manuscript:

- List of abbreviations: The list is incomplete, e.g. Fig. 9E can only be understood when reading the result text in which it is explained that here glucagon secretion is described. I suggest to control the complete text for further missing abbreviations (such as e.g.sys art V or Ce V in Table 1A). Perhaps this part would be easier to read when presented as a table (if this should be allowed by the journal). Some symbols are used in a double sense, e.g. P for pressure and for permeability. Perhaps it would be easier for the reader to use ‘p’ for pressure (in physics ‘P’ stands for power).
- P.9, paragraph about renal glucose excretion: 10 % filtration rate is only a meaningful estimate for glomerular filtration rate if referred to renal artery blood flow (and not renal artery plasma flow, which is the usual reference value for GFR in text books). So perhaps adding the word ‘blood’ might make this estimation more clear.
- Legend of Fig. 2, panel D: should be ‘… decreased aortic pressure and arterial volume’.
- Several figures: please add the units (e.g. mM to Sys glucose in Fig. 4D and so on) to all subheadings of all figures.
- P.26, 3 paragraph: I miss any explanation/discussion for the biphasic insulin response presented in Fig. 10C.

 

 Typographical errors:

- p.8. last paragraph (right): Kirchhoff (not Kirchoff).

I have read this submission. I believe that I have an appropriate level of expertise to confirm that it is of an acceptable scientific standard, however I have significant reservations, as outlined above.
